# Selectivity by host plants affects the distribution of arbuscular mycorrhizal fungi: evidence from ITS rDNA sequence metadata

**DOI:** 10.1186/1471-2148-12-50

**Published:** 2012-04-12

**Authors:** Haishui Yang, Yanyan Zang, Yongge Yuan, Jianjun Tang, Xin Chen

**Affiliations:** 1 Institute of Ecology, School of Life Sciences, Zijingang Campus of Zhejiang University, No 668 of Yuhang Road, Hangzhou, China

## Abstract

**Background:**

Arbuscular mycorrhizal fungi (AMF) can form obligate symbioses with the vast majority of land plants, and AMF distribution patterns have received increasing attention from researchers. At the local scale, the distribution of AMF is well documented. Studies at large scales, however, are limited because intensive sampling is difficult. Here, we used ITS rDNA sequence metadata obtained from public databases to study the distribution of AMF at continental and global scales. We also used these sequence metadata to investigate whether host plant is the main factor that affects the distribution of AMF at large scales.

**Results:**

We defined 305 ITS virtual taxa (ITS-VTs) among all sequences of the Glomeromycota by using a comprehensive maximum likelihood phylogenetic analysis. Each host taxonomic order averaged about 53% specific ITS-VTs, and approximately 60% of the ITS-VTs were host specific. Those ITS-VTs with wide host range showed wide geographic distribution. Most ITS-VTs occurred in only one type of host functional group. The distributions of most ITS-VTs were limited across ecosystem, across continent, across biogeographical realm, and across climatic zone. Non-metric multidimensional scaling analysis (NMDS) showed that AMF community composition differed among functional groups of hosts, and among ecosystem, continent, biogeographical realm, and climatic zone. The Mantel test showed that AMF community composition was significantly correlated with plant community composition among ecosystem, among continent, among biogeographical realm, and among climatic zone. The structural equation modeling (SEM) showed that the effects of ecosystem, continent, biogeographical realm, and climatic zone were mainly indirect on AMF distribution, but plant had strongly direct effects on AMF.

**Conclusion:**

The distribution of AMF as indicated by ITS rDNA sequences showed a pattern of high endemism at large scales. This pattern indicates high specificity of AMF for host at different scales (plant taxonomic order and functional group) and high selectivity from host plants for AMF. The effects of ecosystemic, biogeographical, continental and climatic factors on AMF distribution might be mediated by host plants.

## Background

Arbuscular mycorrhizal fungi (AMF) are widespread in terrestrial ecosystems and form obligatory symbiotic relationships with most land plants [1]. These symbioses are not host specific but are to some extent host-preferential [1]. AMF may have a biogeographical distribution partially because of their soil-borne life form, host preference, and limited dispersal resulting from geographic isolation [2]. Increasingly, studies have shown that AMF are cosmopolitan at the genus or higher taxonomic level but have limited distributions at the species level. Global-scale studies showed that AMF have distinct distribution patterns [3] and that habitat filtering or dispersal limitation affects these patterns [4]. At the continental scale, a single widespread host plant was found primarily to harbor geographically generalist AMF [5]. At the regional scale, a typical distance-decay distribution of AMF suggested that geographic distance and environmental heterogeneity determined the patterns of spatial scaling [6]. At the local scale, AMF distribution is closely related to host identity and habitat [7]. At the fine scale, AMF diversity has a patchy distribution [8]. Studies have also shown that AMF distribution could be influenced by climate [4,9], habitat [7], geographical isolation, soil conditions, and anthropogenic activities [6].

Several studies have reported high selectivity between host plants and AMF, which may affect AMF distribution [10-12]. Bever *et al *[10] reported host-specific differences in the population growth rates of AMF. Helgason *et al *[11] found physical and functional selectivity in AMF, and Zhang *et al. *[12] recently documented that the invasive plant *Solidago canadensis *promoted the growth of the most beneficial AMF and thereby increased its own competitiveness. Distinctive AMF communities were associated with coexisting plant species, including grasses [13,14], forbs [15,16], and trees [17,18]. Even plants of the same species that differed in age harbored distinctive AMF [19,20]. Thus, it seems likely that plants can actively select with AMF to associate. Some studies have suggested that plants have evolved an ability to recognize beneficial AMF. For example, Bever *et al *[21] found that the host plant preferentially allocated carbon to beneficial symbionts. Kiers *et al *[22], who used qPCR and stable isotope probing methods, documented the existence of a reciprocal rewards mechanism that stabilizes the cooperation between plants and AMF. Because host plants have distinct geographical distributions [23], selection by host plants may result in distinct geographical distributions for AMF [24]. However, whether and how host selection affects the pattern of AMF distribution at large scales has not been well studied.

Recently, two studies have revealed global patterns of AMF by using molecular approach. One of these studies [3] has developed a database (MaarjAM) and demonstrated ecosystemic and global patterns for AMF distribution. This study also documented that AMF had high specificity of host plant superorders and wider distribution with larger breadth of hosts. The other study [25] also found a high endemism of AMF at global scales that could be affected by geographic distance, soil condition and plant community. Although both of these studies indicated that host plants related to AMF distribution at a global scale, whether and how host plants affect AMF distribution remain unknown. Here, we hypothesized that selective pressure from host plants at different scales (taxonomic order and functional group) may be the main factor affecting the distribution of AMF at large scales, and that the effects of biogeographical, ecosystemic and climatic factors may be mediated by host plants.

ITS (Internal Transcribed Spacer), SSU (Small Sub-Unit) and LSU (Large Sub-Unit) are the three widely used marker genes in molecular diversity of AMF. SSU was utilized widely while LSU was relatively infrequent compared with ITS (based on Kivlin's comprehensive publication survey [25] and genus search tool of *emerencia *[26] as well as personal survey from GenBank). As relative conserved variability of sequences among species, SSU is widely used in molecular diversity of AMF. However, SSU has a limitation to resolve species in genera of *Ambispora, Diversispora *and *Scutellospora *[27-29]. This limitation can affect the designation of phylotype and further influence the analysis on the distribution of the whole AMF [30]. For LSU, although it had relative higher intra-specific variability than SSU and can also well resolve AMF species, the LSU based data in GenBank are limited for the study of AMF distribution at global scale. To avoid the limitations of SSU and LSU, we selected ITS as marker gene for our analysis. Although ITS has a high intra- and inter-specific variability and is not suitable for operational approaches, it can provide sufficient phylogenetic signal that fit well in the phylogenetic methods. Moreover, the complete ITS region could separate almost all AMF species except *Glomus intraradices *and its close relatives [31]. In addition, abundant ITS sequences are stored in public databases that support our present study. Also, the online genus search tool *emerencia *[26] provides great convenience for extracting all the AMF ITS sequences from GenBank.

In this study, we used data mining to explore the diversity and distribution of AMF based on ITS sequences in public databases. Our objectives were: 1) to explore the global distribution patterns of AMF; 2) to test whether selectivity for host at scales of taxonomic order and functional group is the main factor affecting the current distribution patterns of AMF.

## Results

### AMF ITS-VT determination and phylogeny

**Figure 1:**
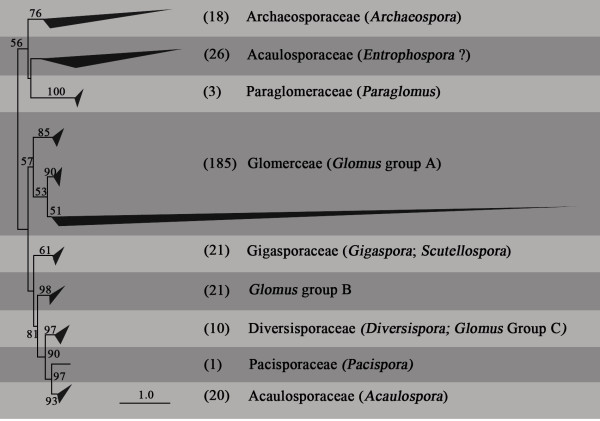
**Phylogenetic relationships of glomeromycotan families**. The phylogeny was constructed by maximum likelihood method with GTRCAT-GAMMA model. Bootstrap values > 50% are shown above the branch.

Using entire ITS sequences of each genus, we delimited ITS-VT (ITS virtual taxa) based on the maximum likelihood neighbor-joining phylogeny and 90% sequence similarity. A total of 305 ITS-VTs were obtained. Some ITS-VTs included named glomeromycotan species, while some named species were grouped into ITS-VTs. The maximum likelihood tree distinguished nine Glomeromycota families (Figure 1). The genus *Glomus *clustered into three distinct clades: clade A (GlGrA), B (GlGrB), and C (GlGrC). GlGrA included three distinct subgroups. Diversisporaceae was composed of GlGrC and *Diversispora*. Similarly, taxa from *Gigaspora *and *Scutellospora *were grouped together in the clade of Gigasporaceae. However, the Acaulosporaceae was represented by two phylogenetically distant clades, one corresponding to *Acaulospora *and one to *Entrophospora*. The *Entrophospora *clade was uncertain. This clade included all the *Entrophospora *ITS-VTs, three *Acaulospora *ITS-VTs, and three *Glomus *ITS-VTs. The three *Glomus *ITS-VTs were singletons, were annotated with uncultured fungi, and were assigned to *Glomus *genus according to *emerencia*, but the maximum similarity with *Glomus *was only about 80% through BLAST.

### AMF ITS-VT richness

**Figure 2:**
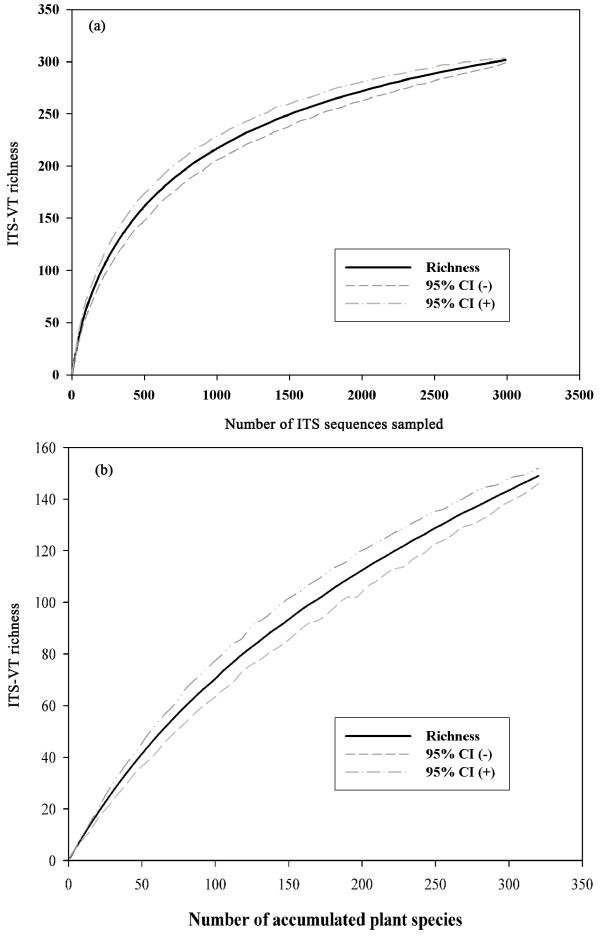
**Rarefaction curves of sampling intensity**. (**a**) The unsaturated relationship between global ITS-VT richness and sampling sequences indicated that more ITS-VTs would be uncovered with increasing sampling. (**b**) The positive near- linear curve suggested quite more ITS-VTs would be found with increasingly sampled host plants. The dashed line indicates the 95% confidence interval.

The rarefaction curve did not reach an asymptote at the global scale, suggesting that some or many taxa have not yet been sequenced (Figure 2a). Meanwhile, the rarefied relationship between accumulated plant species and ITS-VTs showed a positive near-linear curve, indicating that more ITS-VTs would be found with increasingly sampled plants (Figure 2b). Of the 305 ITS-VTs, 70 were singletons and 46 were doubletons. Glomeraceae was dominant and represented by 185 ITS-VTs (Figure 1). The Acaulosporaceae-*Entrophospora *branch was the second largest clade, with 26 ITS-VTs. The third and fourth largest clades were GlGrB and Gigasporaceae, each with 21 ITS-VTs. The Acaulosporaceae-*Acaulospora *branch was the fifth largest clade, with 20 ITS-VTs. Smaller glomeromycotan families included Diversisporaceae with 10 ITS-VTs, Paraglomeracae with 3 ITS-VTs, and Pacisporaceae with 1 ITS-VT (Figure 1; see Additional file 1).

The numbers and percentage of ITS-VTs for each genus are shown in Additional file 2. With respect to genera, *Glomus *was dominant, i.e., *Glomus *was represented by the largest proportion of ITS-VTs for continent, biogeographical realm, climatic zone and supercontinent, totally accounting for 74%. The numbers of genera were highest for Europe and North America among continents, Palearctic and Nearctic regions among biogeographical realms, regions with warm temperature among climatic zones, and Laurasia with super-continent, indicating biased sampling intensity. When the uneven sampling intensity was removed, the ITS-VT richness was not significantly different among plant functional groups, among ecosystems or among super-continents. However, for climatic zones, snow and polar zones had lower ITS-VT richness than other regions (*P *< 0.05); for continents, ITS-VT richness in Asia and South America was lower than in Europe, North America, and Oceania (*P *< 0.05); for biogeographical realms, ITS-VT richness in Neotropic regions was lower than other regions (*P *< 0.05) (see Additional file 3).

### AMF ITS-VT distribution patterns across spatial scales

**Figure 3:**
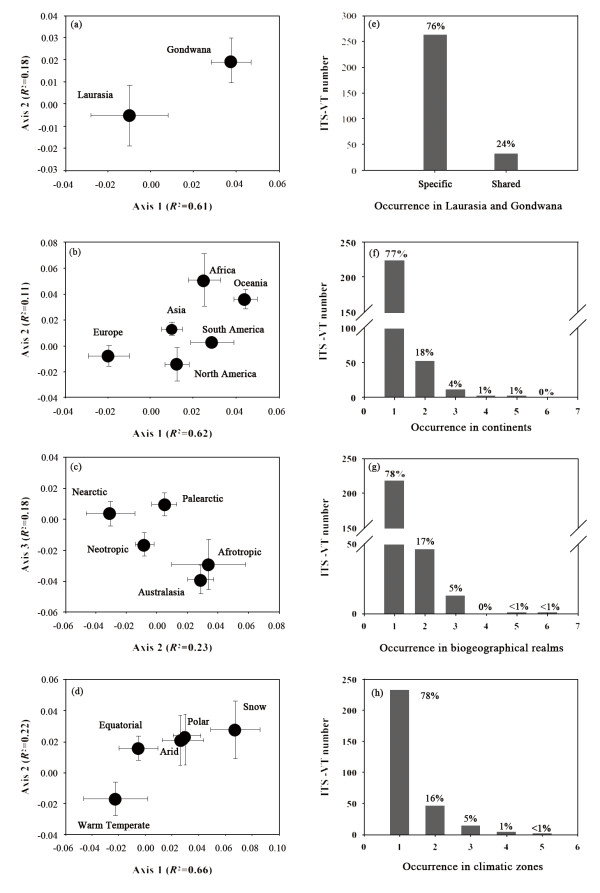
**Comparative composition and specificity of ITS-VTs across spatial provinces: Non-metric multidimensional scaling analysis (NMDS) based on Bray-Curtis method revealed distinct composition of ITS-VTs among Pangaea (**a**), continents (**c**), biogeographical realms (**e**), and climatic zones (**g**)**. High endemism of ITS-VTs was showed across Pangaea (**b**), continents (**d**), biogeographical realms (**f**), and climatic zones (**h**).

The distribution of ITS-VTs was assessed at spatial scales of super-continents, biogeographical realms, continents and climatic zones (Figure 3). Non-metric multidimensional scaling analysis (NMDS) showed distinctive ITS-VT composition between Laurasia and Gondwana (Figure 3a). Across supercontinents, totally 76% ITS-VT specifically occurred in Laurasia or Gondwana, while a small proportion of 24% were shared (Figure 3b). Across continents, NMDS revealed distinct ITS-VT composition in Europe, North America, Asia, and South America, but not evidently different between Africa and Oceania (Figure 3c). 77% ITS-VTs were unique to one continent (Figure 3d). At the scale of biogeographical realm, Palearctic, Nearctic and Neotropic had distinctive ITS-VT composition, but not for Afrotropic and Australasia (Figure 3e). 78% ITS-VTs occurred in one type of biogeographical realms (Figure 3f). At the scale of climatic zones, evidently different ITS-VTs occurred in warm temperate regions compared with other zones (Figure 3g). High endemism was shown with specific occurrence of 78% at the scale of climatic zones (Figure 3h).

### Host plant distribution patterns across spatial scales

**Figure 4:**
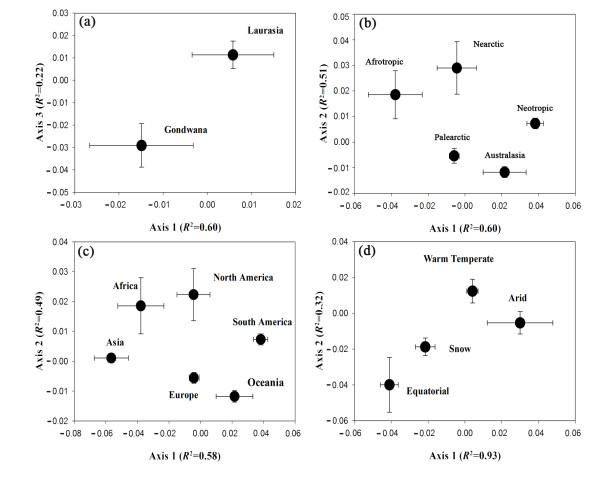
**Comparative composition of host plant taxonomic order across spatial provinces**. Non-metric multidimensional scaling analysis (NMDS) based on Bray-Curtis method showed distinct composition of host plant taxonomic order across Pangeae (**a**), biogeographical realms (**b**), continents (**c**) and climatic zones (**d**).

Non-metric multidimensional scaling analysis (NMDS) showed that spatial factors could drive host plant distributions (Figure 4). AMF associated with plant taxonomic orders were totally different between Laurasia and Gondwana (Figure 4a). At the scale of biogeographical realms, Palearctic, Neotropic and Australasia had distinct AMF based on plant order composition, but not for Nearctic and Afrotropic (Figure 4b). Similar results were shown across continents (Figure 4c). In addition, the AMF associated with taxonomic orders of plants was distinctive among different climatic zones (Figure 4d).

### Host selectivity at the scales of ecological groups and the distribution for ITS-VTs

**Figure 5:**
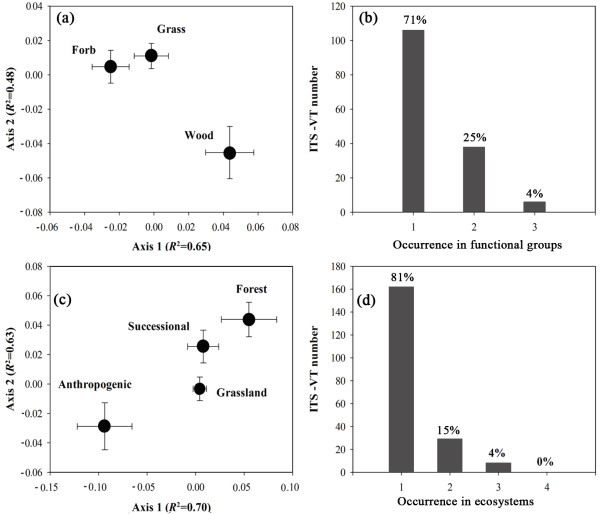
**Comparative composition and specificity of ITS-VTs across biotic groups**. Non-metric multidimensional scaling analysis (NMDS) based on Bray-Curtis method revealed distinct composition of ITS-VTs among host plant functional groups (**a**) and ecosystems (**c**); High specific occurrence of ITS-VTs across functional groups of hosts (**b**) and ecosystems (**d**).

AMF host selectivity at the scale of ecological group (functional groups and ecosystems) affected the distribution of AMF (Figure 5). NMDS revealed that AMF ITS-VT composition was totally different among grass, forb and wood (Figure 5a). About 71% ITS-VTs were unique to one type of functional group while only a small proportion of 4% ITS-VTs were shared by all three functional groups (Figure 5b). At the scale of ecosystems, grassland, forest and anthropogenic system had distinctive AM fungal communities; while AMF community in successional system was not evidently different with grassland and forest (Figure 5c). In addition, high ecosystemic specificity was found and the specific ITS-VTs occurring in a single ecosystem accounted for 81%. No ITS-VT was shared by four types of ecosystems (Figure 5d).

### Host selectivity at the scale of plant taxonomic order and the distribution for AMF ITS-VTs

**Figure 6:**
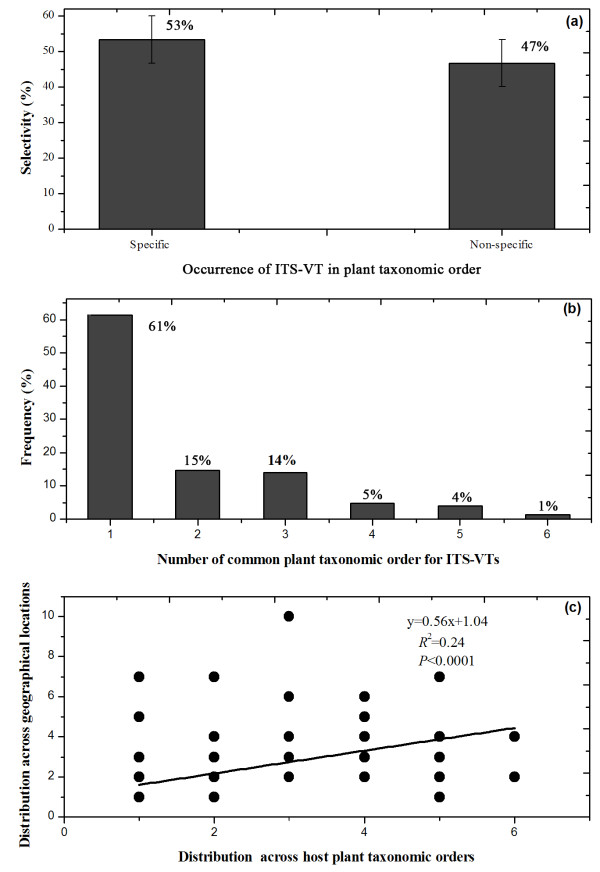
**The relationship between ITS-VT distribution and selectivity from plant hosts**. (**a**) Host plant taxonomic orders had high specific selectivity for ITS-VT communities; (**b**) The occurrence of ITS-VTs was high specific across host plant taxonomic orders; (**c**) These ITS-VTs with higher occurrence among host taxonomic orders had wider geographical distributions.

Host plant order had high specific selectivity for AMF and each taxonomic order had 53% specific ITS-VTs (Figure 6a); meanwhile, ITS-VTs showed high host specificity (Figure 6b). Most ITS-VTs were associated with only one host taxonomic order, and only a small proportion of ITS-VTs occurred simultaneously in 6 host orders (Figure 6b).

Linear regression analysis showed a positive relationship between the distribution of AMF across plant taxonomic order and across geographical locations (Figure 6c). ITS-VT geographical distribution increased as host range increased (*R*^2 ^= 0.24, *P *< 0.001).

**Figure 7:**
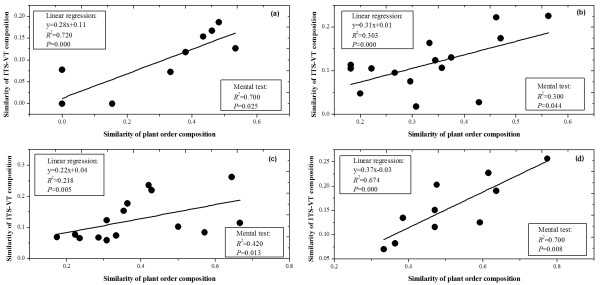
**The relationship between the distribution of plant taxonomic order and ITS-VT across ecosystems (a), biogeographical realms (b), continents (c) and climatic zones (d)**. Linear regression analysis showed that ecosystem (**a**), biogeographical realm (**b**), continent (**c**) and climatic zone (**d**) with more similar plant composition had more similar ITS-VT composition. Mantel test suggested plant composition significantly correlated to ITS-VT composition among each type of biomes.

Meanwhile, according to linear regression analysis, compositional similarity of plant taxonomic order and ITS-VT was significantly correlated across ecosystems (Figure 7a, *R*^2 ^= 0.72, *P *= 0.000), biogeographical realms (Figure 7b, *R*^2 ^= 0.300, *P *= 0.044), continents (Figure 7c, *R*^2 ^= 0.218, *P *= 0.005) and climatic zones (Figure 7d, *R*^2 ^= 0.674, *P *= 0.000). Mantel test also showed that AMF distribution was significantly related to the distribution of plant taxonomic orders across ecosystem (*R*^2 ^= 0.700, *P *= 0.025), biogeographical realms (*R*^2 ^= 0.300, *P *= 0.044), continents (*R*^2 ^= 0.420, *P *= 0.013) and climatic zones (*R*^2 ^= 0.700, *P *= 0.008) (Figure 7a-d).

**Figure 8:**
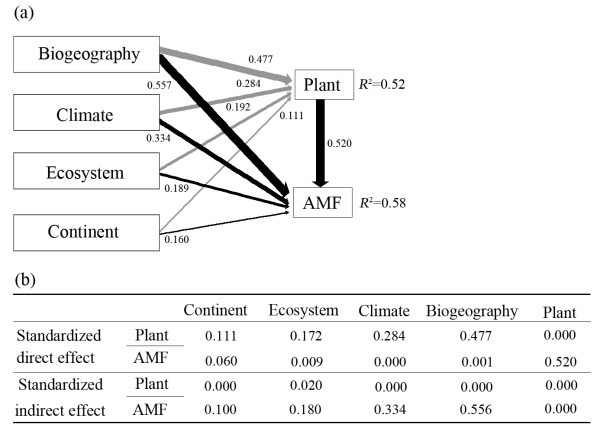
**Ecosystem, climate, continent and biogeographical realm influence directly or indirectly the distribution of plants and AMF**. (**a**) A structural equation model showing the standardized total effects of ecosystemic, climatic, continental and biogeographical factors on plant distribution and the standardized total effects of ecosystemic, climatic, continental and biogeographical factors and host plants on AMF distribution. (**b**) Standardized direct and indirect effects of factors mentioned above on plant and AMF distribution. The numbers above the arrows represented path coefficients, and the width of arrows indicated the strength of the causal effects.

Our data fit the SEM analysis (χ^2 ^= 6.524, *P *= 0.771; *RMSEA *= 0.048; *TLI *= 0.971). SEM indicated that ecosystem, climate, biogeography and continent directly affected the distribution of plants, and indirectly affected the distribution of AMF (Figure 8). These factors accounted for 52% variance for plant distribution and 59% variance for AMF distribution. SEM showed that biogeography and plant were the two most important factors (with path coefficient of 0.557 for biogeography and 0.520 for host plant) that affected AMF distribution (Figure 8a). However, biogeography had lower direct effect (with path coefficient of 0.001) but higher indirect effect (with path coefficient of 0.556) on AMF. Plant had strong direct effects (with path coefficient of 0.520) but no indirect effects on AMF distribution (with path coefficient of 0.000). In addition, the effects of climate, ecosystem and continent on plant distribution were direct, while these effects on AMF distribution were indirect (Figure 8b).

## Discussion

### Identification of ITS-VTs

In this study, we defined 305 ITS-VTs of AMF based on maximum likelihood phylogenetics for each genus. Our phylogenetic reconstruction of all ITS-VTs was different from earlier studies [32,33]. The genus *Glomus *was grouped into three distinct clades (GlGrA, GlGrB, and GlGrC), and GlGrA contained three subgroups. GlGrA, GlGrB, and GlGrC were distantly related to each other; meanwhile, GlGrC was placed in the Diversisporaceae clade. This polyphyletic pattern was consistent with classical taxonomic identification and description [32,33]. GlGrB was distantly related to GlGrA in our analysis. This is not consistent with a previous phylogenetic reconstruction based on the SSU gene [33]. This difference may result from different evolutionary rates of AMF clades for the ITS and SSU gene. In addition, the phylogenetic placement of Acaulosporaceae remains controversial. According to Schüßler *et al *[32], Acaulosporaceae consists of *Acaulospora *and *Entrophospora*, but in the current study, *Acaulospora *and *Entrophospora *were phylogenetically separated at the family level, not at the genus level. This might result from the misidentification of the *Entrophospora *morphospecies that were the sources of the original ITS reference sequences. Meanwhile, three *Glomus *ITS-VTs occurred in the Acaulosporaceae-*Entrophospora *clade. These ITS-VTs were singletons, annotated with uncultured fungus and had low sequence similarity with *Glomus *species. This might be errors of *emerencia*. In addition, previous studies [32,33] suggested *Acaulospora *and *Entrophospora *were two genera in the family of Acaulosporaceae. However, both clades were phylogenetically distant at the family level. Therefore, it also seems possible that a new family should be defined for *Entrophospora *clade.

### High local diversity of AMF and selectivity of host plants

Our results showed that AMF have high local diversity and that most AMF have narrow distribution ranges. These patterns might be explained by high selectivity of AMF by their hosts. The selectivity from host plants could occur at different scales, such as plant taxonomic levels of species, family and order as well as functional groups and plant communities. In our study, about 60% of ITS-VTs were host specific. 71% ITS-VTs occurred in a single functional group. Only a small proportion of ITS-VTs had broad host ranges and functional group ranges.

Host selectivity for AMF could result in host plants harboring distinctive AMF and explain high AMF diversity at the local scale. Vandenkoornhuyse *et al *[14] found that coexisting grass species had distinct AMF communities. Martinez-Garcia *et al *[15] reported high host preference of AMF in semiarid environments. In oak woodland, Douhan *et al *[17] found contrasting AMF communities associated with three commonly occurring plants. Similar results were also obtained from trees in tropical forests [18]. Findings in these studies are consistent with the idea that hosts might exert selective pressure on AMF communities [13] and influence AMF local diversity. Alguacil *et al *[24] documented that plant types promoted AMF diversity. Johnson *et al *[34] found that plant communities could significantly affect AMF diversity and composition. In addition, interactions among host plants might also drive development of specific AMF communities [35]. Thus, a host plant diversified community could result in high local diversity of AMF.

### Effects of host plants on the distribution of AMF

This study showed that AMF exhibited distinct ecosystemic, climatic, biogeographical and continental patterns (Figure 3), and these patterns were correlated with host plant distribution (Figure 7). For ecosystems, Kivlin et al [25] reported that AMF had an ecosystemic distribution pattern, and argued this distribution was a confounding host plant effect. In this study, AMF distribution was significantly correlated with host plant distribution across ecosystems (Figure 7a) and our SEM also showed that ecosystems could directly influence plant distribution but indirectly affect AMF distribution (Figure 8). Meanwhile, SEM revealed host plant had strong direct effects on AMF. Therefore, AMF ecosystemic pattern might be mediated by host plants. Ecosystems consisted of distinctive plants, e.g., crop plants were limited to anthropogenic systems and most trees only occurred in forests; this is leading to high specificity of AMF for certain ecosystems and distinct ecosystemic composition because of high host specificity of AMF (Figure 6). In addition, specific interactions between plants in certain ecosystems might exert direct effects on the ecosystemic pattern of AMF [35].

Our results showed that AMF had high specificity and distinct composition for climatic zones (Figure 3g and 3f). We determined that climatic factors maybe affect AMF distribution. However, our SEM suggested that climate had no direct effect but relatively stronger indirect effects on AMF (Figure 8b). The indirect effects on AMF might be through plants and ecosystems because of strong host effects (Figure 8a). Studies had showed that climate was a main driving factor for plant distribution and different climatic zones had distinct ecosystems [36]. The selectivity from host plants and ecosystems might form the current distribution pattern of AMF in climatic zones.

In addition, our study revealed high endemism and distinct distribution pattern of AMF across continents (Figure 3c-d). Our results also showed that plant had a continent distribution pattern and closely correlated to AMF distribution. Our SEM further showed that continent had strong indirect effect but weak direct effect on AMF (Figure 8b). This indicated that the continental limitation for AMF distribution might be a function of plant distribution. Long- range dispersal of host plants across ocean is relative uncommon, but plants dispersal within continents occurs more frequently. Dispersal limitation of AMF exists among continents but is not significant within continents for AMF which was reported by Kivlin et al [25].

Previous studies suggested historical factors could affect the distributions of AMF associated with plants and AMF at global scale [3,25,37]. In this study, the paleocontinents, Laurasia and Gondwana showed significantly different AMF composition (Figure 3a-b). The AMF composition among biogeographical realms was also distinctive (Figure 3e-f). Our SEM showed that biogeography had a strong direct effect on plant distribution but a strong indirect effect on AMF distribution (Figure 8b). It suggested the effects of biogeography might be mediated by plants. From geological study [38], Laurasia separated from Gondwana at Triasic of Mesozoic (251-199.6 Mya); at early Cretaceous (145.5-65.5 Mya), Gondwana departed into Afrotropic, Oceania, Indo-Malay and Neotropic; and Laurasia finally divided into Palearctic and Nearctic at 55 Mya. Evidently, the Ordovician origin (460 Mya) of AMF far predated the separation of Laurasia and Gondwana as well as the formation of current continents [2]. Thus, we could assume that AMF was randomly distributed and formed a 'common pool' at Pangaea; as Pangaea separated into Laurasia and Gondwana, AMF composition in these two supercontinents would be similar. However, our results and previous study [3] showed that only a small proportion of AMF was shared by Laurasia and Gondwana. Two possibilities could attribute to this phenomenon. One is that new speciation of AMF occurred after the division of Laurasia and Gondwana and after the separation of the current tectonic plates. This could have been mediated by host plants. At late Cretaceous, angiosperm plants explosively appeared [39], resulting in strong selective pressure to promote AM fungal speciation. Another possibility was that there were far more AMF in the 'common pool' than present. After large numbers of angiosperm plants speciated, climate and other natural selective pressures would promote the revolution of different plants on different tectonic plates. Because of asymmetric and obligatory nature of mycorrhiza, some AMF associated with compatible host, and co-evolved to form present patterns, while others did not and went extinction.

Although our results suggest that host plant directly affects AMF, climatic, ecosystemic, continental and biogeographical factors also indirectly influence the distribution of AMF, all of these factors can only explain 58% variance of AMF distribution. There is still 42% variance waiting for explanation. This might be the effects of soil, because soil conditions have been found to significantly affect AMF distribution [25].

## Conclusions

This study shows that host plants have high selectivity for AMF and that AMF have high host specificity and local diversity. AMF with low host specificity have a wide geographical distribution, but most AMF have a restricted distribution, i.e., most have been only found in ecosystems, or in biogeographical realms, or in climatic zones, or in continents. Consequently, AMF have distribution patterns across ecosystems, across biogeographical realms, across climatic zones and across continents. Plant composition is significantly correlated with AMF composition. Ecosystems, biogeographical realms, climatic zones, and continents had relatively stronger direct effects on plants than indirect effects, but stronger indirect effects on AMF than direct effects. Biogeography and plant are the two strongest factors on AMF, but the effect of biogeography is indirect while direct for plant. It suggested the effects of ecosystems, biogeographical realms, climatic zones and continents on AMF distribution might be mediated by plants through high host selectivity.

## Methods

### Data source

The ITS sequence data of glomeromycotan fungi were extracted from GenBank with the genus search tool in *emerencia *[26]. The insufficiently identified ITS sequences (IIS) were extracted with *emerencia *from 71 published and 30 unpublished studies (see Additional file 4). The sufficiently identified ITS sequences (FIS) were from 44 published and 21 unpublished studies (see Additional file 5). The sequences were then processed with FungalITSextractor software [40]. Only the whole ITS region (ITS1-5.8 S-ITS2) was used for further analysis. Details of each study were compiled, including sample origin (roots, soil, or spore), biotic factors (order, family, genus, species and functional group of host plants, and ecosystem), and spatial distribution (geographical coordinate, site, continent, biogeographical realm, climatic zone, and latitudinal zone). For the unpublished studies, if the annotation information in GenBank was insufficient, the author was contacted. For the origin of cultured fungi, the public Glomeromycota collection databases (BEG, INVAM, GINCO) were checked, and the authors were also contacted if necessary. The glomeromycotan nomenclature follows Arthur Schüßler's *Glomeromycotan *phylogeny [http://www.lrz.de/~schuessler/amphylo/; 25 July 2011]. Host plant order follows APG III [41].

### Data collection

With the online genus search tool *emerencia*, we obtained at total of 3547 ITS sequences from GenBank, including 2236 insufficiently identified sequences (IIS) and 544 sufficiently identified sequences (FIS) from *Glomus*, 114 IIS and 64 FIS from *Acaulospora*, 84 IIS and 7 FIS from *Archeaospora*, 18 FIS from *Diversispora*, 29 IIS and 15 FIS from *Entrophospora*, 22 IIS and 153 FIS from *Gigaspora*, 116 IIS and 20 FIS from *Paraglomus*, 50 IIS and 73 FIS from *Scutellospora*, and 2 IIS from *Pacispora*. After processing with FungalITSextractor, sub-regions of ITS (such as only ITS1 or ITS2) were excluded, and 3055 whole ITS regions (ITS1-5.8 S-ITS2) were obtained, including 2464 ITS from *Glomus*, 173 from *Acaulospora*, 58 from *Archeaospora*, 18 from *Diversispora*, 25 from *Entrophospora*, 103 from *Gigaspora*, 125 from *Paraglomus*, 87 from *Scutellospora*, and 2 from *Pacispora*. The whole ITS regions were used for further analysis. Of the 3055 ITS sequences in this study, 1809 were from plant roots, and the paired data of ITS-VT and plant identity were used for these analyses. A total of 90 host plant species were included in this study, and these belonged to 70 genera, 32 families, and 29 orders. However, the 90 plant species only occurred in one location of one continent, and no one plant species were shared by continents, this might lead to distinctive AMF communities among plants at species level. Therefore, we used data at plant taxonomic order level for further analysis on the selectivity and distribution of AMF.

### Data analysis

#### Phylogenetic analysis

For analyzing the IIS sequences from *Glomus *genus, we used a two-step method. First, the sequences were clustered into operational taxonomic units (OTU) based on 97% similarity. This was done with Mothur version 1.19.0 [42]. With the 'get.oturep' command, one representative sequence for each OTU was selected. Because of the high variability in the ITS region of glomeromycotan fungi, 97% similarity is insufficient to distinguish a taxon; therefore, representative sequences were grouped further with a maximum likelihood tree based on 50% bootstrap support (see Additional file 6 Additional file 7). The phylogenetic analysis was conducted in RAxML version 7.0 with the 'GTRCAT' model [43]. For these sequences clustered on the phylogenetic tree, but with less than 50% bootstrap support, 90% similarity was used to determine the assignment of these sequences (see Additional file 6 Additional file 7). This similarity cutoff was inferred from FIS sequences used in this study (see Additional file 5) with Mothur [42]. In keeping with Öpik's virtual taxa (VT) [3], we defined these sequence groupings as 'ITS-VT'. A representative sequence for each ITS-VT was randomly selected, and picked randomly from FIS of well-identified AMF species; and phylogenetic relationships of all ITS-VTs (including IIS and FIS) from *Glomus *were reconstructed with the maximum likelihood method by the 'GTRCAT' model in RAxML version 7.0 [43]. For identification of ITS-VTs from *Acaulospora, Entrophospora, Archaeospora, Gigaspora, Scutellospora*, and *Paraglomus*, we used maximum likelihood phylogenetic method based on 50% bootstrap support and 90% similarity for sequence groups less than 50% bootstrap support (see Additional file 8 Additional file 9 Additional file 10 Additional file 11 Additional file 12 and Additional file 13). This ITS-VT identification based on phylogenetics did not include Diversispora and Pacispora, because these two genera only had one species respectively. Randomly picked-representatives of ITS-VT from each genus were used to reconstruct the phylogenetic relationships of AMF based on maximum likelihood method with the models of 'GTRCAT', 'GTRGAMMA', 'GTRCAT-GAMMA' and 'GTRGAMMAI' respectively. All the phylogenetic trees were made from the online display and annotation tool *iTOL *[44].

#### Spatial effect analysis

Four kinds of spatial provinces were recognized: continents, super-continents, biogeographical realms, climatic regions. The continents were defined as Africa, North America (including Latin America), South America, Oceania, Europe, and Asia. Following Scotese's suggestion [38], we also considered Laurasia and Gondwana, which were created when the hypothetical supercontinent of Pangaea broke in two and then eventually formed the continents that currently exist: Laurasia included present-day North America and Eurasia, while Gondwana consists of Africa, South America, Oceania, and India. Following Olsen *et al *[45], we distinguished eight biogeographical realms: Palearctic, Nearctic, Afrotropic, Neotropic, Indo-Malay, Australasia, Oceania, and Antarctic. In this study, no information was obtained for Oceania or Antarctic. Because only one sample was found in Indo-Malay, the data were not used in the further statistic and multivariate analysis. According to the Köppen-Geiger world climatic map [46], we classified climatic regions into five types: arid, equatorial, polar, snow, and warm temperature. We analyzed the occurrence, richness and composition of ITS-VTs as well as plant composition in each of the four kinds of spatial province.

#### Host plant effect analysis

We analyzed the relationship between host plants and the distribution of glomeromycotan fungi at the level of host taxonomic order, host functional group, and ecosystem. First, host selectivity for ITS-VTs was evaluated at the taxonomic order level. We defined the proportion of specific ITS-VTs to total ITS-VTs harbored by one host taxonomic order as host plant selectivity. The specific occurrence of ITS-VTs across host taxonomic order was also evaluated. Here, we defined the distribution of ITS-VT across hosts as the occurrence of ITS-VTs in each host order. We classified functional groups as grasses, forbs, and woody plants, and evaluated the occurrence and composition of ITS-VTs in these functional groups. We evaluated the distribution of ITS-VTs among five types of ecosystems, and these were anthropogenic systems (mainly for agricultural systems), forests, grasslands, shrublands, and successional systems (mainly for wetlands). Because of small samples in shrublands (involving one sample and two host plants), this ecosystem was not included in the further analysis.

#### Statistical analysis

Rarefaction analysis was performed by the randomization algorithm incorporated in Ecosim version 7.0 [47] with 1000 iterations, and an accumulative curve was generated to show the relationship between ITS-VT richness and sampling intensity, and between ITS-VT richness and sampled plant species.

Linear regression analysis was performed in SPSS 16.0 (SPSS Inc., USA) to test whether the distribution of ITS-VT across host taxonomic order was related to that across geographical locations. Meanwhile, linear regression analysis was carried out to elucidate the relationship between the composition similarity of ITS-VTs and plant taxonomic orders in ecosystems, biogeographical realms, continents and climatic zones.

The non-metric multidimensional scaling (NMDS) analysis was used to compare AMF composition in ecological groups (functional groups and ecosystems) and spatial provinces (continents, biogeographical realms, super-continent, and climatic zones). For analysis of functional groups and ecosystems with NMDS, each plant was used as one replication. Plants harboring 3 or more ITS-VTs were used in order to keep intensive sampling. For analysis of spatial provinces with NMDS, each location was used as one replication. Location but not each study was used in the analysis in keep of sufficient sampling with at least 12 DNA sequence or 6 or more ITS-VTs. Location was defined based on administrative province and ecosystem type. This analysis was executed in Past, version 1.91 [48], with the Bray-Curtis similarity method. NMDS was also used to elucidate the distribution patterns of plant at taxonomic order level across ecosystems and spatial provinces with Bray-Curtis method.

In order to differentiate the effects of original geographical distribution of AMF with host plant drivers, first, Mantel test was used to determine whether AMF distribution was correlated with plant distribution among ecosystems and spatial provinces (with XLSTAT 7.5: Addinsoft Inc., France). Then, we used the structural equation modeling (SEM) to decipher the direct and indirect effects of these factors of plants, ecosystem, climate, biogeography and continent on AMF distribution. The distribution of AMF and plant were represented by the scores of the first component of PCA of the composition of ITS-VTs and plant taxonomic order among sampling locations. Similarly, the first component scores of PCA of categorical composition of ecosystem, biogeographical realm, continent and climatic zone were used as the variables of ecosystem, biogeography, continent and climate. This method was referred with Liu et al [49] and the analysis was performed in Amos 17.0 (Smallwaters Corporation, Chicago, IL, USA).

## Competing interests

The authors declare that they have no competing interests.

## Authors' contributions

HY and XC had the main responsibility for the study and participated in all parts of it, including development of the main idea, study setup, collection of a small portion of the data, data analysis, manuscript preparation, and manuscript revision. YZ and YY collected most of the data. JT was responsible for data interpretation and also assisted with manuscript revision. All authors read and approved the final manuscript.

## Supplementary Material

Additional file 1 **Figure S1. The details of the maximum likelihood collapsed tree of all ITS-VTs with GTRCAT_GAMMA model**. Figure S1. Bootstrap values > 50% are shown above the branch. (PDF 227 kb). Click here for file

Additional file 2 **Figure S2 Number and proportion of ITS-VT number of each genus**. (a) among continents, (b) among biogeographical realms, (c) among climatic zones and between Laurasia and (d) Gondwana. The number in parenthesis was total number of ITS-VTs. (PDF 212 kb). Click here for file

Additional file 3 **Figure S3 ITS-VT richness among host functional groups, ecosystems, climatic zones, continents, biogeographical realms and supercontinent**. (a) ITS-VT richness in different host functional groups: forbs, grasses, and woody plants. (b) ITS-VT richness in different ecosystems: anthropogenic ecosystem, forest, grassland, and successional ecosystem. (c) ITS-VT richness in different climatic zones: arid, equatorial, polar, snow and warm temperate; (d) ITS-VT richness in different continents: Europe, Asia, North America, Africa, South America and Oceania; (e) ITS-VT richness in different biogeographical realms: Palearctic, Nearctic, Afrotropic, Neotropic and Australasia; (f) ITS-VT Fig. S1Laurasia and Gondwana. (TIFF 221kb). Click here for file

Additional file 4 **Table S1. Details of insufficiently identified ITS (IIS) data source used in this study (PDF 173kb)**. Click here for file

Additional file 5 **Table S2. Details of sufficiently identified ITS (FIS) data source used in this study (PDF 190kb)**. Click here for file

Additional file 6 **Figure S4 Identification of ITS-VTs from IIS of Glomus genus with maximum likelihood method**. ITS-VTs were determined with representative IIS sequence clusters (97% similarity) based on 50% support value; for sequence clusters under 50% bootstrap support, further 90% similarity was used to assign these sequences into ITS-VTs. (PDF 530) Click here for file

Additional file 7 **Figure S5 Maximum likelihood tree showed the phylogenetic relationship between ITS-VTs from IIS and FIS of Glomus**. Bootstrap values > 50% are shown above the branch. (PDF 41 kb). Click here for file

Additional file 8 **Figure S6 Identification of ITS-VTs from IIS and FIS of Entrophospora genus with maximum likelihood method**. ITS-VTs were determined with sequence groups based on 50% bootstrap support; for sequence clusters under 50% bootstrap support, further 90% similarity was used to assign these sequences into ITS-VTs. (PDF 210 kb). Click here for file

Additional file 9 **Figure S7 Identification of ITS-VTs from IIS and FIS of Paraglomus genus with maximum likelihood method**. ITS-VTs were determined with sequence groups based on 50% bootstrap support; for sequence clusters under 50% bootstrap support, further 90% similarity was used to assign these sequences into ITS-VTs. (PDF 212 kb). Click here for file

Additional file 10 **Figure S8 Identification of ITS-VTs from IIS and FIS of Archaeospora genus with maximum likelihood method**. ITS-VTs were determined with sequence groups based on 50% bootstrap support; for sequence clusters under 50% bootstrap support, further 90% similarity was used to assign these sequences into ITS-VTs. (PDF 225 kb). Click here for file

Additional file 11 **Figure S9 Identification of ITS-VTs from IIS and FIS of Scutellospora genus with maximum likelihood method**. ITS-VTs were determined with sequence groups based on 50% bootstrap support; for sequence clusters under 50% bootstrap support, further 90% similarity was used to assign these sequences into ITS-VTs. (PDF 227 kb). Click here for file

Additional file 12 **Figure S10 Identification of ITS-VTs from IIS and FIS of Acaulospora genus with maximum likelihood method**. ITS-VTs were determined with sequence groups based on 50% bootstrap support; for sequence clusters under 50% bootstrap support, further 90% similarity was used to assign these sequences into ITS-VTs. (PDF 241 kb). Click here for file

Additional file 13 **Figure S11 Identification of ITS-VTs from IIS and FIS of Gigaspora genus with maximum likelihood method**. ITS-VTs were determined with sequence groups based on 50% bootstrap support; for sequence clusters under 50% bootstrap support, further 90% similarity was used to assign these sequences into ITS-VTs. (PDF 209 kb). Click here for file
