# Supplementary figures and images for: Selectivity by host plants affects the distribution of arbuscular mycorrhizal fungi: evidence from ITS rDNA sequence metadata
Source: BMC Evol Biol. 2012 Apr 12;12:50. doi: 10.1186/1471-2148-12-50 (PMC3395829; doi:10.1186/1471-2148-12-50)

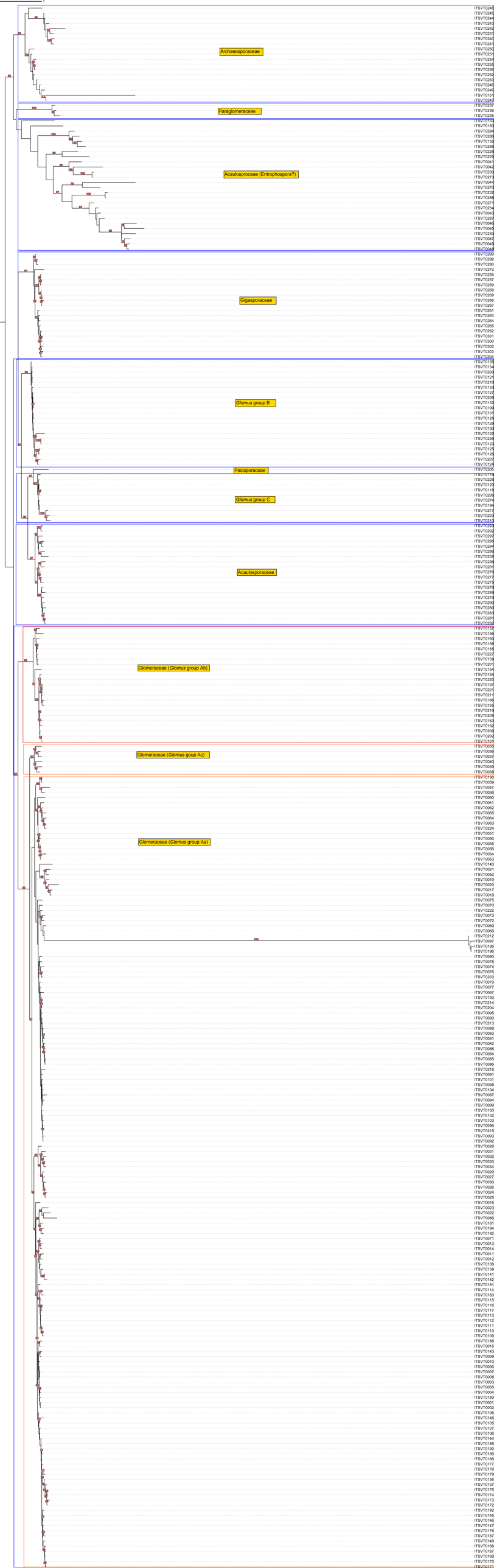

Supplement: Additional file 1 — Figure S1. The details of the maximum likelihood collapsed tree of all ITS-VTs with GTRCAT_GAMMA model. Figure S1. Bootstrap values > 50% are shown above the branch. (PDF 227 kb). [file 1471-2148-12-50-S1.PDF]

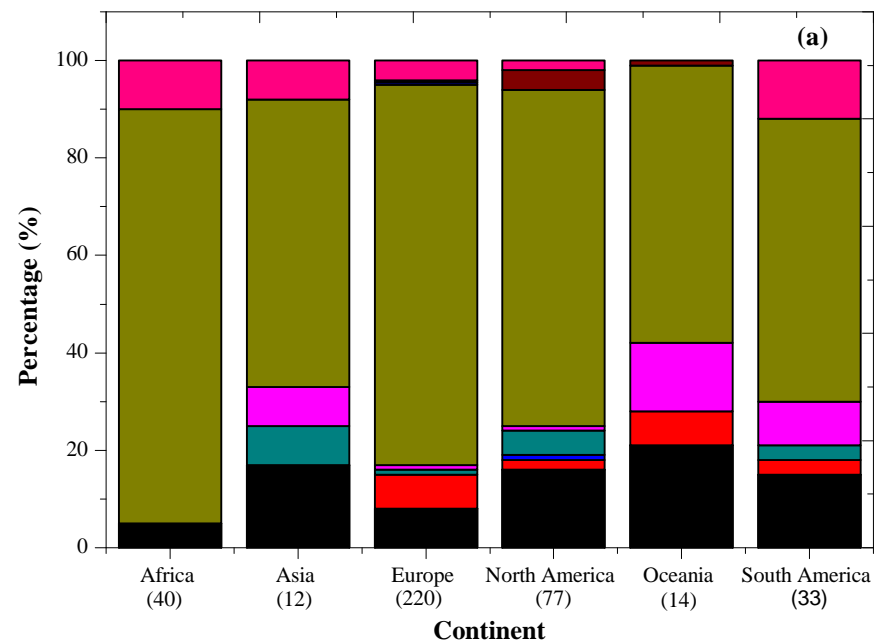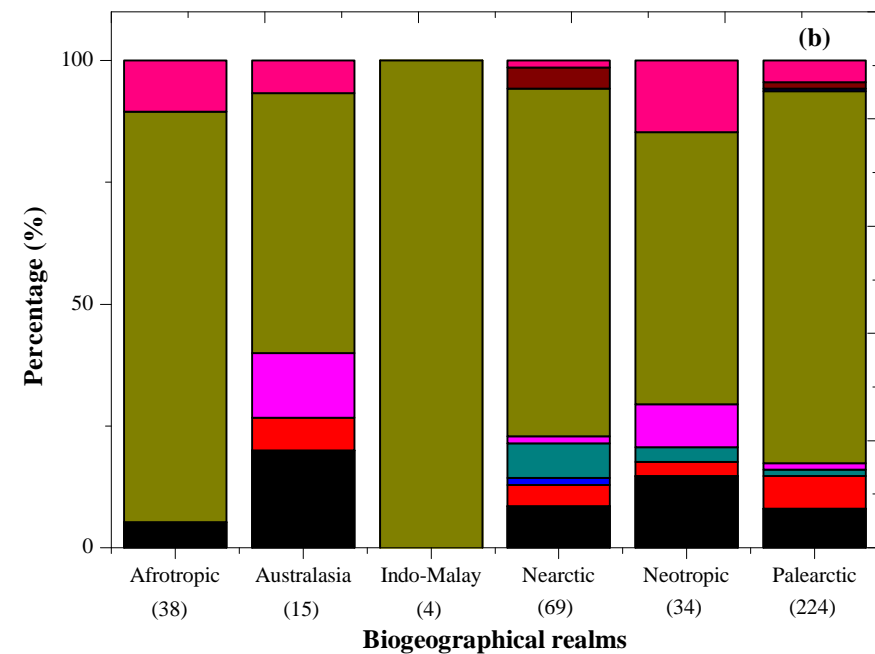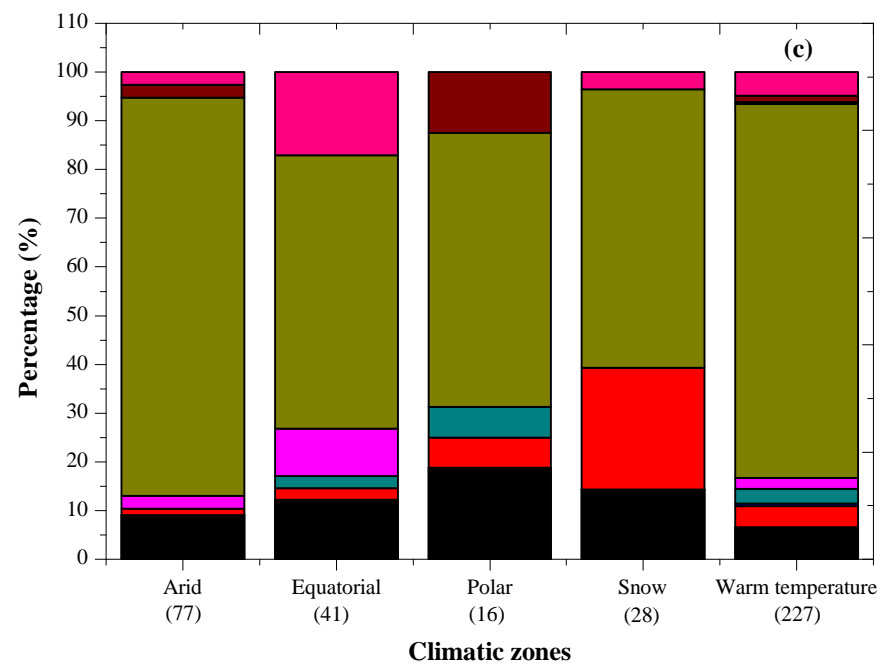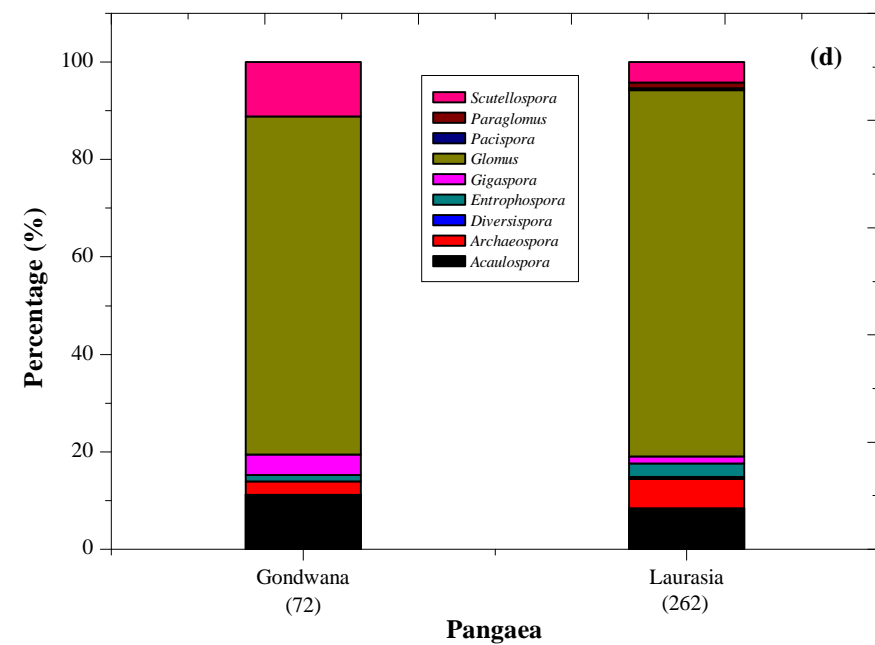

Supplement: Additional file 2 — Figure S2 Number and proportion of ITS-VT number of each genus. (a) among continents, (b) among biogeographical realms, (c) among climatic zones and between Laurasia and (d) Gondwana. The number in parenthesis was total number of ITS-VTs. (PDF 212 kb). [file 1471-2148-12-50-S2.PDF]

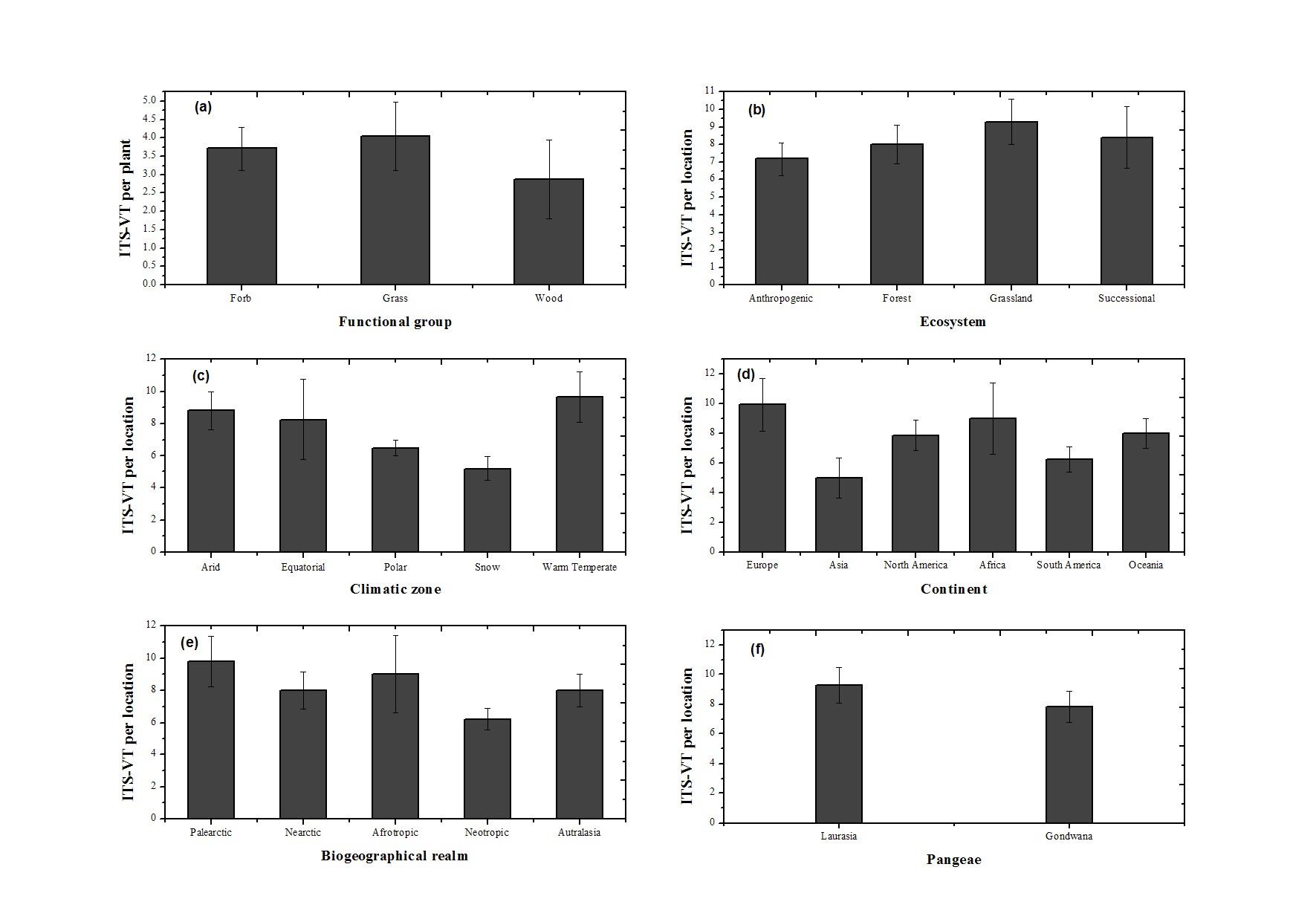

Supplement: Additional file 3 — Figure S3 ITS-VT richness among host functional groups, ecosystems, climatic zones, continents, biogeographical realms and supercontinent. (a) ITS-VT richness in different host functional groups: forbs, grasses, and woody plants. (b) ITS-VT richness in different ecosystems: anthropogenic ecosystem, forest, grassland, and successional ecosystem. (c) ITS-VT richness in different climatic zones: arid, equatorial, polar, snow and warm temperate; (d) ITS-VT richness in different continents: Europe, Asia, North America, Africa, South America and Oceania; (e) ITS-VT richness in different biogeographical realms: Palearctic, Nearctic, Afrotropic, Neotropic and Australasia; (f) ITS-VT Fig. S1Laurasia and Gondwana. (TIFF 221kb). [file 1471-2148-12-50-S3.TIFF]

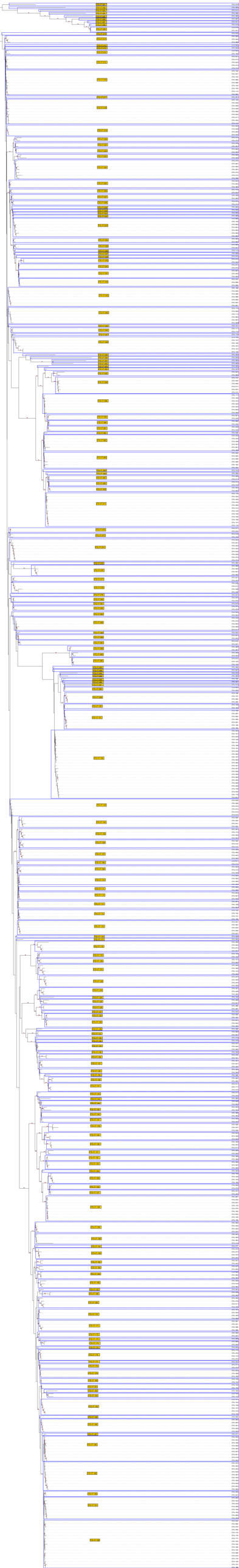

Supplement: Additional file 6 — Figure S4 Identification of ITS-VTs from IIS of Glomus genus with maximum likelihood method. ITS-VTs were determined with representative IIS sequence clusters (97% similarity) based on 50% support value; for sequence clusters under 50% bootstrap support, further 90% similarity was used to assign these sequences into ITS-VTs. (PDF 530) [file 1471-2148-12-50-S6.PDF]

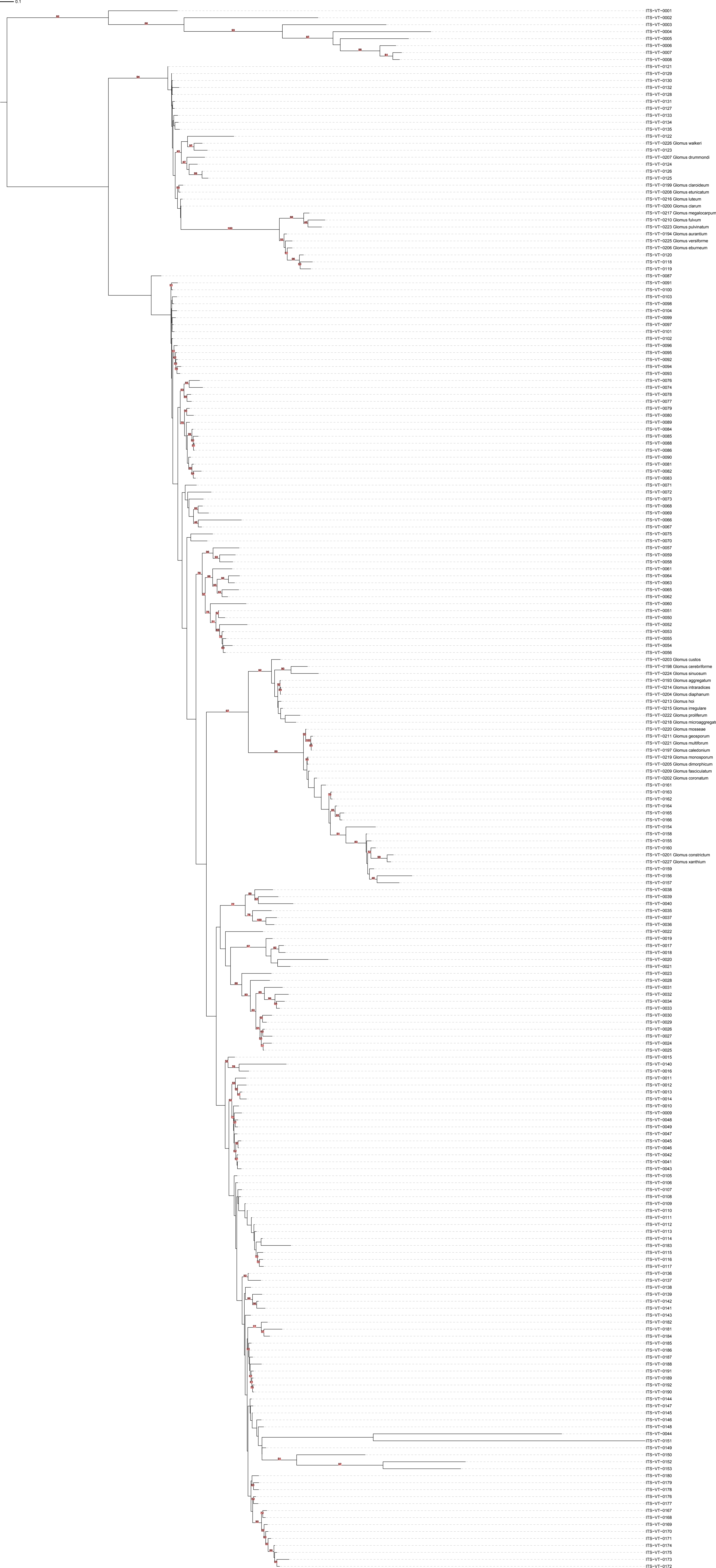

Supplement: Additional file 7 — Figure S5 Maximum likelihood tree showed the phylogenetic relationship between ITS-VTs from IIS and FIS of Glomus. Bootstrap values > 50% are shown above the branch. (PDF 41 kb). [file 1471-2148-12-50-S7.PDF]

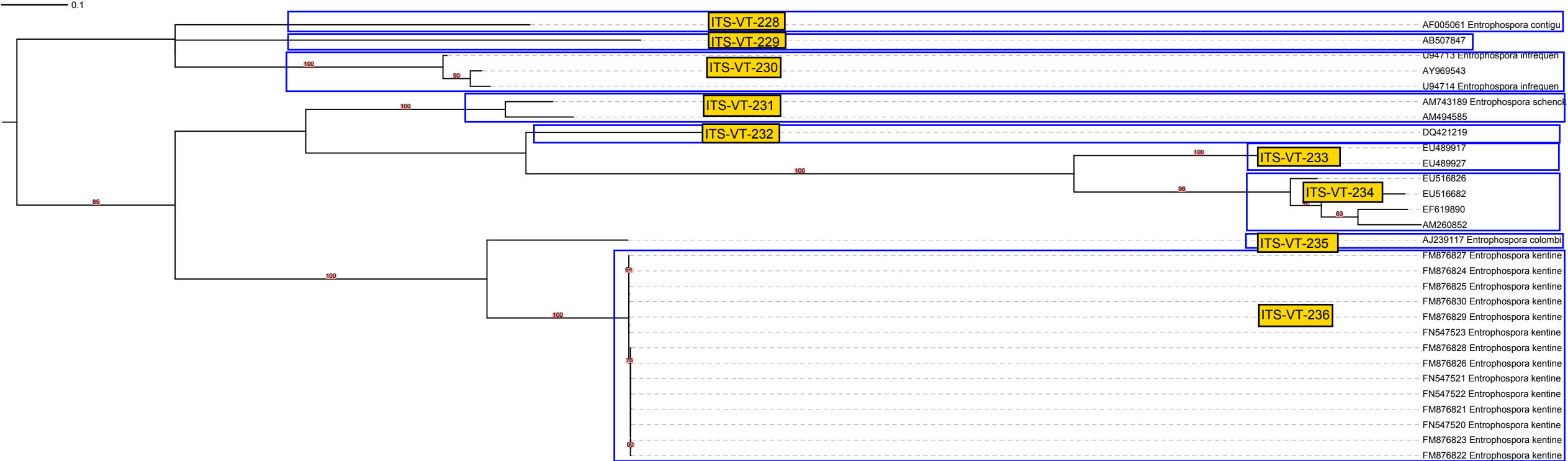

Supplement: Additional file 8 — Figure S6 Identification of ITS-VTs from IIS and FIS of Entrophospora genus with maximum likelihood method. ITS-VTs were determined with sequence groups based on 50% bootstrap support; for sequence clusters under 50% bootstrap support, further 90% similarity was used to assign these sequences into ITS-VTs. (PDF 210 kb). [file 1471-2148-12-50-S8.PDF]

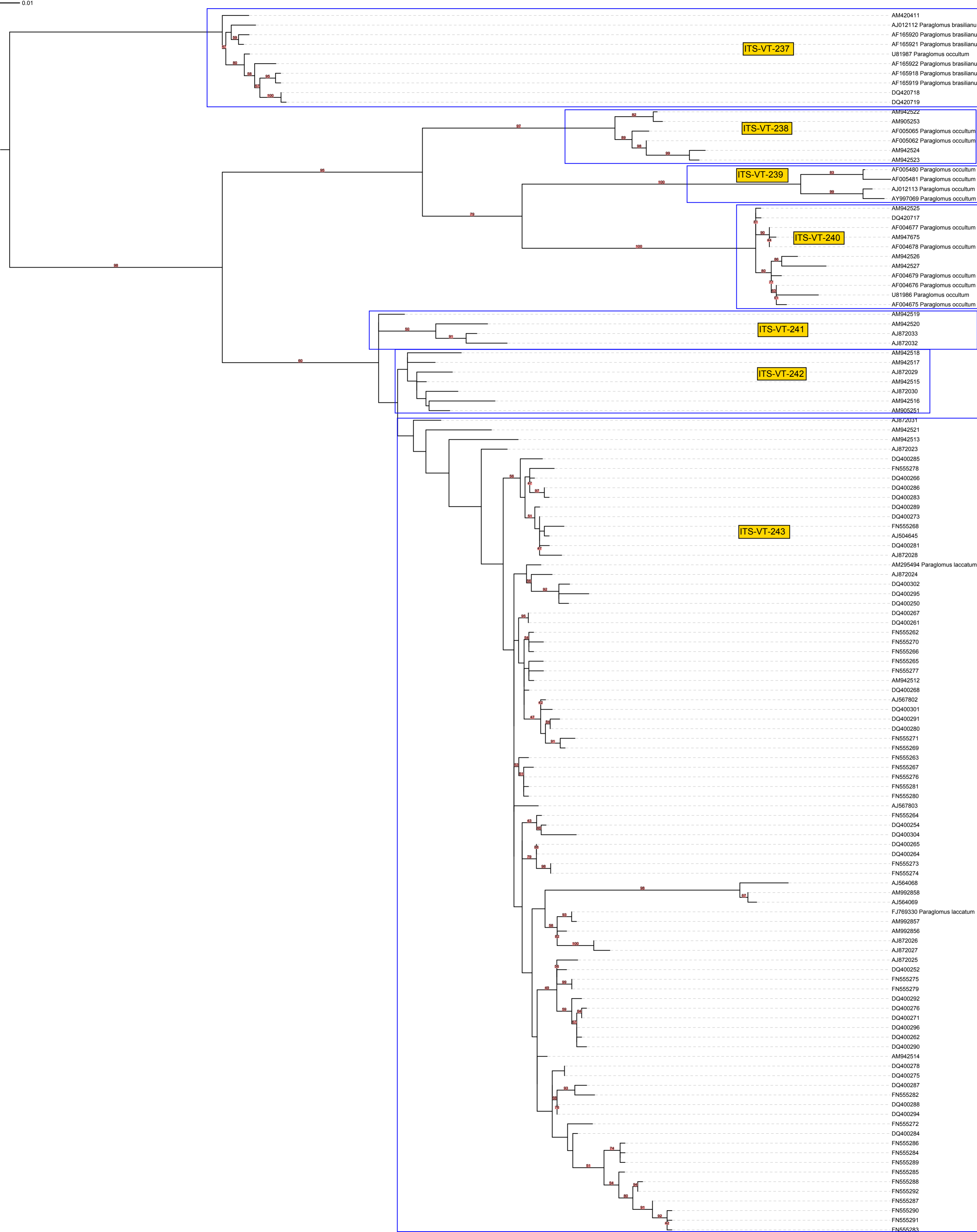

Supplement: Additional file 9 — Figure S7 Identification of ITS-VTs from IIS and FIS of Paraglomus genus with maximum likelihood method. ITS-VTs were determined with sequence groups based on 50% bootstrap support; for sequence clusters under 50% bootstrap support, further 90% similarity was used to assign these sequences into ITS-VTs. (PDF 212 kb). [file 1471-2148-12-50-S9.PDF]

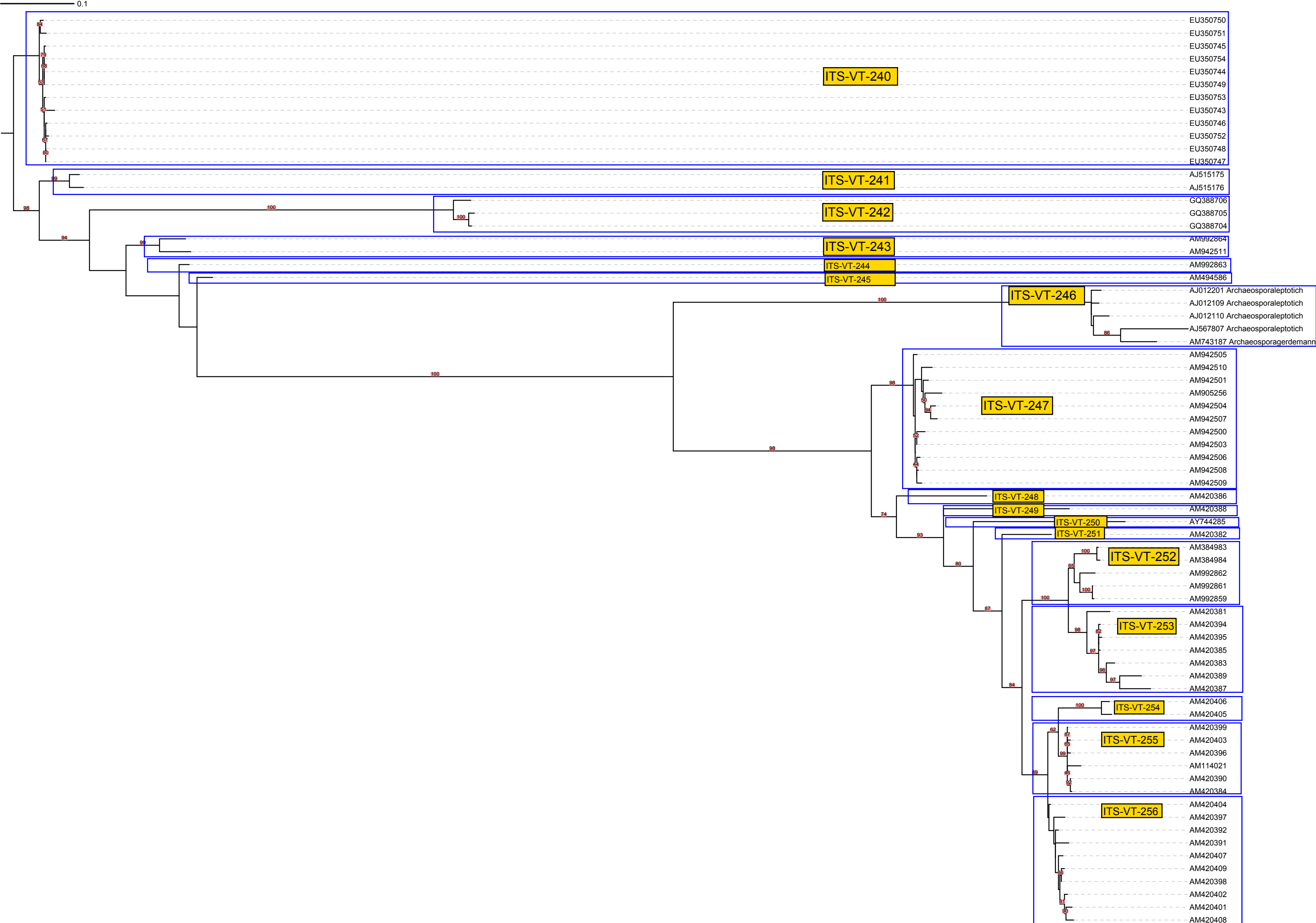

Supplement: Additional file 10 — Figure S8 Identification of ITS-VTs from IIS and FIS of Archaeospora genus with maximum likelihood method. ITS-VTs were determined with sequence groups based on 50% bootstrap support; for sequence clusters under 50% bootstrap support, further 90% similarity was used to assign these sequences into ITS-VTs. (PDF 225 kb). [file 1471-2148-12-50-S10.PDF]

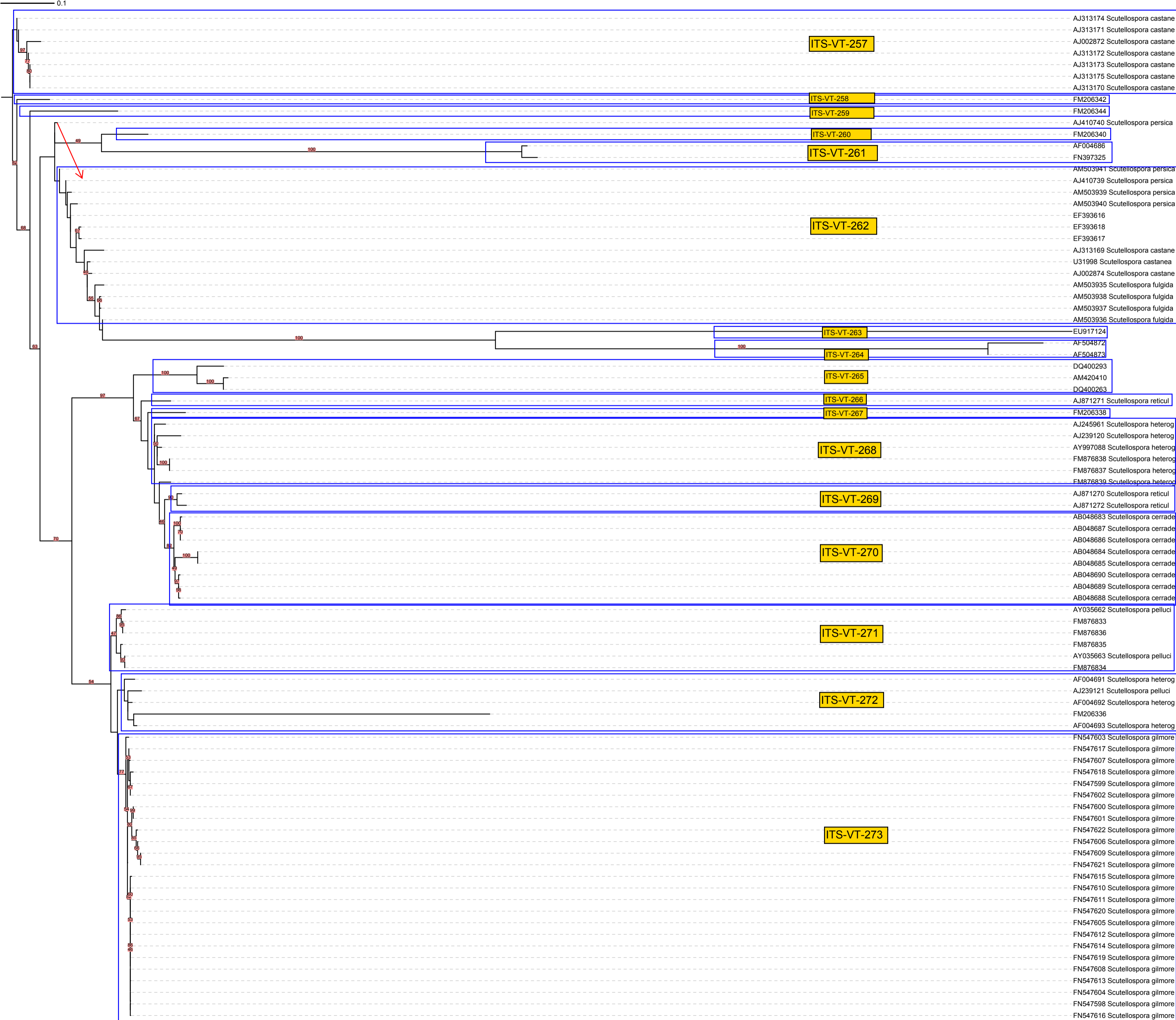

Supplement: Additional file 11 — Figure S9 Identification of ITS-VTs from IIS and FIS of Scutellospora genus with maximum likelihood method. ITS-VTs were determined with sequence groups based on 50% bootstrap support; for sequence clusters under 50% bootstrap support, further 90% similarity was used to assign these sequences into ITS-VTs. (PDF 227 kb). [file 1471-2148-12-50-S11.PDF]

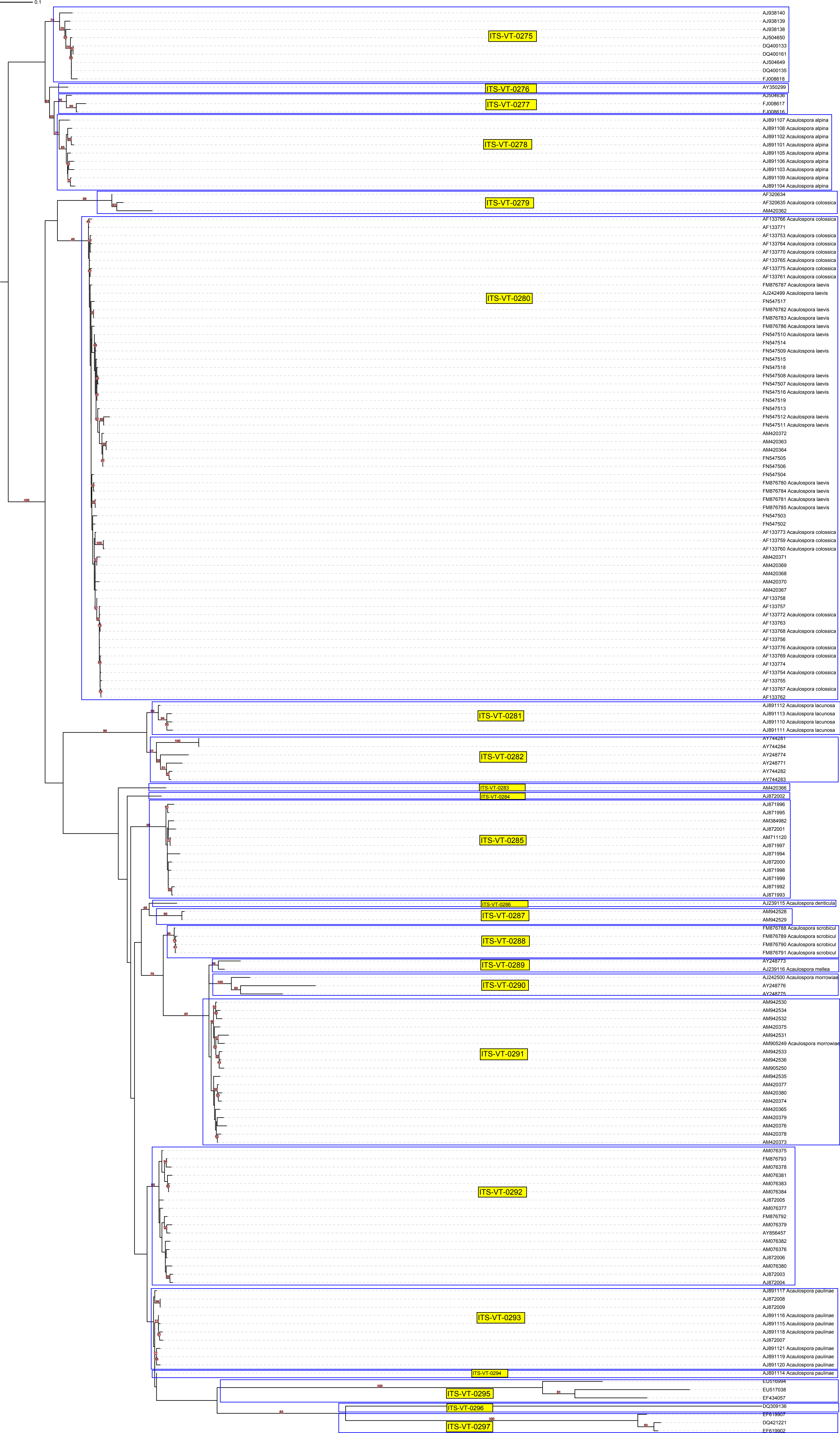

Supplement: Additional file 12 — Figure S10 Identification of ITS-VTs from IIS and FIS of Acaulospora genus with maximum likelihood method. ITS-VTs were determined with sequence groups based on 50% bootstrap support; for sequence clusters under 50% bootstrap support, further 90% similarity was used to assign these sequences into ITS-VTs. (PDF 241 kb). [file 1471-2148-12-50-S12.PDF]

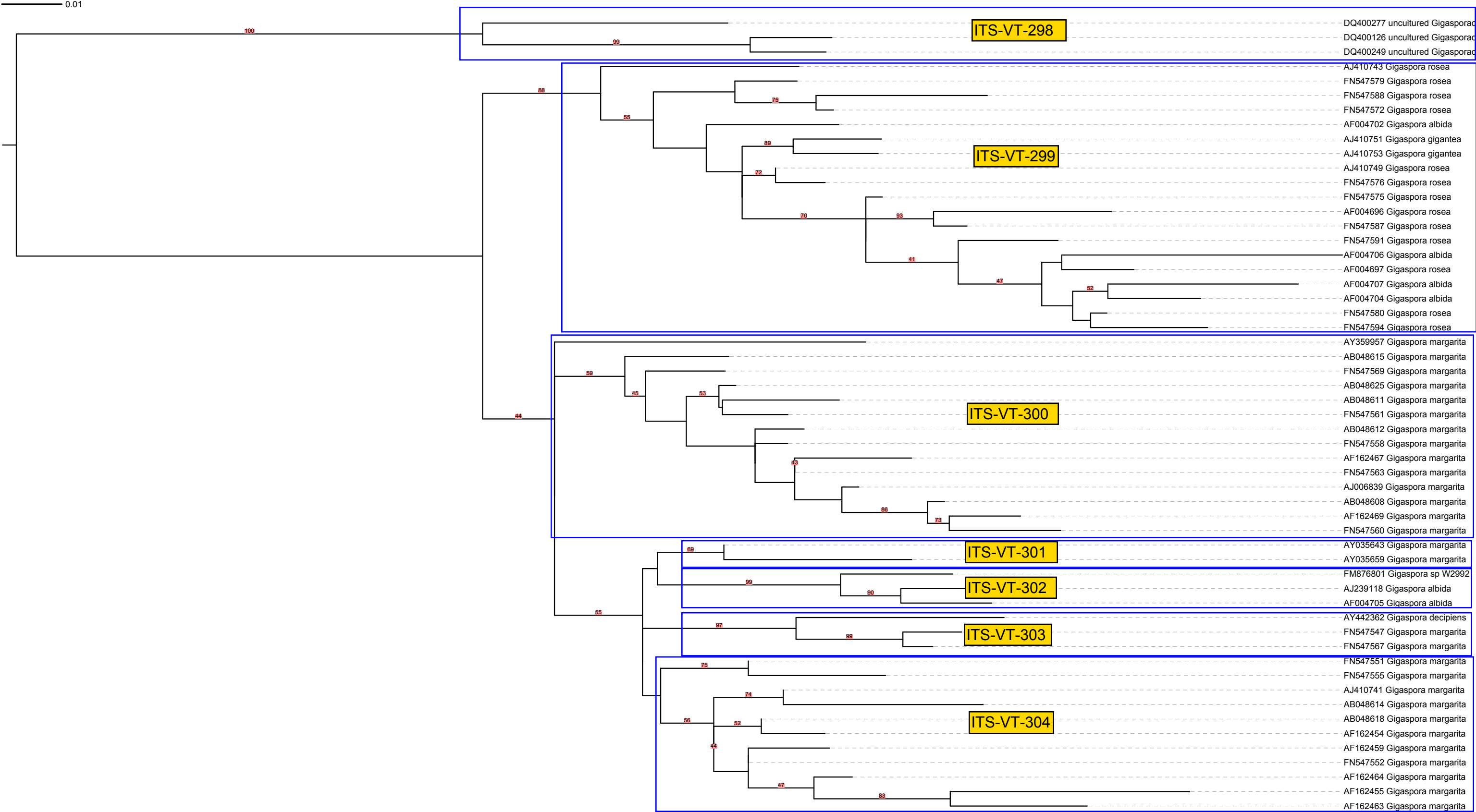

Supplement: Additional file 13 — Figure S11 Identification of ITS-VTs from IIS and FIS of Gigaspora genus with maximum likelihood method. ITS-VTs were determined with sequence groups based on 50% bootstrap support; for sequence clusters under 50% bootstrap support, further 90% similarity was used to assign these sequences into ITS-VTs. (PDF 209 kb). [file 1471-2148-12-50-S13.PDF]
