# Supplementary material for: Selectivity by host plants affects the distribution of arbuscular mycorrhizal fungi: evidence from ITS rDNA sequence metadata
Source: BMC Evol Biol. 2012 Apr 12;12:50. doi: 10.1186/1471-2148-12-50 (PMC3395829; doi:10.1186/1471-2148-12-50)
Supplement: Additional file 4 — Table S1. Details of insufficiently identified ITS (IIS) data source used in this study (PDF 173kb). [file 1471-2148-12-50-S4.PDF]

Table S1 Details of insufficiently identified ITS data source used in this study (to be continued)

Source Reference	Host plant				Sampling site				
	Species	Family	Order	Functional group	Site	Country	Latitude	Longitude	Latitude class
[1]	*Cephalanthera longifolia*	Orchidaceae	Asparagales	forb	Montpellier	France	43.6	3.87	L3
[2]	Soil					Canada	45.26	-75.92	L4
[3]	Soil				Sourhope	UK	55	-3	L4
[4]	3 species [a]	Poaceae; Poaceae;	Cyperales; Poaceae;	grass	Wyoming	US	44.42	-110.61	L3
[5]	Soil				Middlemuir	UK	57.6	-2.16	L4
[6]	10 species [b]	Plantaginaceae; Poaceae; Asteraceae; Rosaceae; Fabaceae	Lamiales; Poales; Asterales; Rosales; Fabales	Forb; Grass	Thuringia	Germany	50.95	11.61	L4
[7]	Freshwater lake				Barstadvatn	Norway	58.51	-6.27	L4
[7]-1	Freshwater lake				Beuven	Netherlands	51.4	5.62	L4
[8]	*Arachnitis uniflora*	Corsiaceae	Liliales	forb	Nahuel Huapi	Argentina	-41.12	-71.392	L3
[9]	Soil				Unknown	Israel	Unknown	Unknown	Unknown

[10]	Soil				Basel	Switzerland	54	15	L4
[11]	Soil				California	USA	38.51	-121.7	L3
[12]	Soil				North Carolina	USA			
[13]	*Epacris pulchella*	Ericaceae	Ericales	wood	Ridgecrop	Australia	-33.7	149.99	L3
[14]	*Calluna vulgaris*	Ericaceae	Ericales	wood	Ridgecrop	Australia	-33.7	149.99	L3
[15]	*Dichanthelium lanuginosum*	Poaceae	Poales	Grass	Wyoming	US	44.41	-110.8	L3
[16]	*Cynara cardunculus var. scolymus*	Asteraceae	Asterales	Forb	Pisa	Italy	43.68	10.31	L3
[17]	Soil				New South Wales	Australia	-33.81	149.95	L3
[18]	*Agathosma betulina*	Rutaceae	Sapindales	Wood	Western Cape	South Africa	-32.76	149.99	L3
[19]	Spore				Tänikon	Switzerland	47.5	8.9	L4
[20]	Soil				Queensland	Australia	-16.52	145.27	L2
[21]	Soil				Alaska	US	68.63	-149.57	L5
[22]	Soil				Saint-Maurice	France	40	-0.5	L3
[23]	*Allium cepa* L.	Amaryllidaceae	Asparagales	Forb	Zeeland	Netherlands	52.4	5.67	L4
[24]	Soil				Zurich	Swiss	47.45	8.49	L4
[25]	*Allium cepa* L.	Amaryllidaceae	Asparagales	Forb	England	UK	52.2	-1.6	L4
[26]	Soil				Massachusetts	US	42.49	-72.19	L3
[27]	Soil				Skulow	Canada	52.32	-121.9	L4
[28]	*Graffenrieda emarginata*	Melastomataceae	Myrtales	Wood	Consuelo	Ecuador	-1.82	-78.2	L1
[29]	Soil				Southwest	Sweden	56.7	13.4	L4

[30]	Mixed roots				Thuringia	Germany	50.95	11.61	L4
[31]	*Zea mays*	Poaceae	Poales	Grass	Basel	Switzerland	47.43	7.75	L4
[31]-1	*Zea mays*	Poaceae	Poales	Grass	Baden-Wuerttemberg	Germany	50.63	10.78	L4
[32]	*Zea mays*	Poaceae	Poales	Grass	Basel	Switzerland	47.43	7.75	L4
[33]	Soil					US	33.86	-99.45	L2
[34]	*Cephalanthera longifolia*	Orchidaceae	Asparagales	Forb	Pussa	Estonia	58.23	22	L4
[35]	*Quercus macrocarpa*	Fagaceae	Fagales	Wood	Kansas	US	39	96	L3
[36]	*Quercus macrocarpa*	Fagaceae	Fagales	Wood	Kansas	US	39	96	L3
[37]	Soil				Thuringia	Germany	50.95	11.61	L4
[38]	*Bouteloua gracilis*	Poaceae	Poales	Grass	New Mexico	US	34.6	-106.67	L3
[39]	Soil				Thuringia	Germany	50.95	11.61	L4
[40]	Soil				Central	Finland	62.17	27.29	L5
[41]	*Botrychium virginianum*	Ophioglossaceae	Ophioglossales	Forb	Kunfeherto	Hungary	47.3	20.21	L4
[42]	Soil					Switzerland	46	8	L4
[43]	*Picea glauca*	Pinaceae	Pinales	Wood	Québec	Canada	52	73	L4
[44]	Soil				Apaj	Hungary	47	19	L4
[45]	*Fraxinus excelsior*	Oleaceae	Lamiales	Wood	Thuringa	Germany	51	10	L4
[46]	*Zea mays*	Poaceae	Poales	Grass	Tsholotsho	Zimbabwe	-19.65	27.82	L2
[47]	Soil				West Virginia	US	38	-80	L3
[48]	Soil				Mariana	Japan	11.35	142.2	L1
[49]	Soil				Mariana	Japan	11.35	142.2	L1
[50]	Soil				Cahors	France	44.26	1.26	L3
[51]	*Phragmites australis*	Poaceae	Poales	Grass	Mainau Bay	Germany	47.7	9.2	L4

[52]	Soil				Durham	US	35.97	-78.93	L3
[53]	Soil				Rotmoos-ferner	Austria	47	14	L4
[54]	Soil				Rotmoos-ferner	Austria	47	14	L4
[55]	Soil				Basel	Switzerland	47.43	7.75	L4
[56]	*Crocus sp*;<br>*Bromus sp*.;<br>*Gentiana acaulis*	Iridaceae<br>Poaceae<br>Gentianaceae	Liliales<br>Poales<br>Gentianales	Forb<br>Grass	Ramosch	Switzerland	46	10	L4
[57]	Soil				Minas Gerais	Brazil	-19.82	-43.95	L2
[58]	Soil				Duke Forest	US	35.97	-78.93	L3
[59]	*Bouteloua gracilis*	Poaceae	Poales	Grass	New Mexico	US	34.4	-106.67	L3
[60]	Soil				Ontario	France	48	-80	L4
[61]	Soil				Duke University	US	35	-78	L3
[62]	Soil				Basel	Switzerland	47	7	L4
[63]	Spore				Sylvia, Florida	US	26.17	-80.17	L2
[64]	14 species [c]	Asteraceae<br>Fabaceae<br>Apiales<br>Rubiaceae<br>Onagraceae<br>Poaceae<br>Araceae	Asterales<br>Fabales<br>Apiaceae<br>Gentianales<br>Myrtales<br>Poales<br>Cyperales<br>Alismatales	Forb<br>Grass	Thuringia	Germany	51	11.67	L4
[65]	14 species [d]	Poaceae;<br>Brassicaceae	Poales;<br>Brassicales	Forb<br>Grass	Thuringia	Germany	51	11.67	L4

		Fabaceae<br>Ranunculaceae<br>Araceae<br>Rubiaceae<br>Onagraceae<br>Asteraceae	Fabales<br>Ranunculales<br>Alismatales<br>Gentianales<br>Myrtales<br>Asterales						
[66]	*Plantago lanceolata*	Plantaginaceae	Lamiales	Forb	Thuringia	Germany	51	11.67	L4
[67]	*Marchantia foliacea*	Marchantiaceae	Marchantiales	Forb	Port Levy	New Zealand	-51.27	1.07	L4
[68]	*Arnica montana*	Asteraceae	Asterales	Forb	Krakow	Poland	50.05	19.93	L4
[69]	4 species [g]	Asteraceae<br>Poaceae<br>Lamiaceae<br>Fabaceae	Asterales<br>Poales<br>Lamiales<br>Fabales	Forb<br>Grass	Alsace	France	47.5	7.5	L4
[70]	9 species [h]	Gentianaceae<br>Iridaceae<br>Lamiaceae<br>Asteraceae<br>Fabaceae<br>Ranunculaceae<br>Asteraceae<br>Polygalaceae	Gentianales<br>Liliales<br>Lamiales<br>Asterales<br>Fabales<br>Ranunculales<br>Asterales<br>Fabales	Forb	Engadin	Switzerland	46.85	10.38	L4
[71]	*Triticum aestivum*	Poaceae;	Poales;	Grass	Tehran	Iran	35.68	51.42	L3
[72]	legume	Fabaceae	Fabales	Forb	Cerrado	Brazil	-24.07	-49.95	L2

[73]	*Triticum aestivum*	Poaceae;	Poales;	Grass	Uttar Pradesh	India	27.57	80.08	L2
[74]	5 species [e]	Fabaceae;<br>Meliaceae	Fabales;<br>Sapindales	Wood	San Vito	Costa Rica	9	-83	L1
[75]	4 species [f]	Fabaceae;<br>Euphorbiaceae<br>Bignoniaceae	Fabales;<br>Euphorbiales<br>Lamiales	Wood	San Vito	Costa Rica	9	-83	L1
[76]	Soil				Quebec	Canada	52	-73	L4
[77]	Apple seedling				New York	US	40	-73	L3
[78]	Soil				Canar	Spain	28	-15.5	L2
[79]	Spore				Basel	Switzerland	47	7	L4
[80]	Spore				Florida	US	28.55	-81.73	L2
[81]	Spore				California	US	38.53	-121.7	L3
[82]	unknown				unknown	Hungary	unknown	unknown	unknown
[83]	Soil				Alaska	US	64	-147	L5
[84]	Soil				Alaska	US	64	-147	L5
[85]	*Dioscorea rotundata*	Dioscoreaceae	Dioscoreales		Southern Guinea	Benin	8.43	2.4	L1
[86]	Spore				Therwil	Switzerland	47.48	7.55	L4
[87]	7 species [j]	Poaceae	Poales	Grass	Mutompo	Namibia	-8.3	19.25	L1
[88]	4 species [i]	Poaceae<br>Fabaceae	Poales<br>Fabales	Grass<br>Wood	Munessa	Namibia	-27	17.5	L2
[89]	Soil				Minnesota	US	44	-93	L3
[90]	*Acer macrophyllum*	Sapindaceae	Sapindales	Wood	Oregon	US	44.5	-121.62	L3
[91]	3 species [k]	Asteraceae<br>Poaceae<br>Amaranthaceae	Asterales<br>Poales<br>Caryophyllales	Forb<br>Grass	Terschelling	Netherlands	53.4	5.4	L4

[91]-1	Mixed Roots				Schreyahn	Germany	52.92	11.07	L4
[93]	*Caltha palustris*	Ranunculaceae	Ranunculales	Forb	Utah	US	39	111	L3
[92]	7 species [l]	Lycopodiaceae	Lycopodiales	Forb	Quito	Ecuador	-0.22	-78.5	L1
[94]	*Dactylis glomerata*	Poaceae	Poales	Grass	Constance	Germany	47.65	9.32	L4
[95]	Soil				Florida	US	27.43	-80.33	L2
[96]	*Taxus baccata*	Taxaceae	Pinales	Wood	Balingen	Germany	48.28	8.82	L4
[97]	*Prunus africana*	Rosaceae	Rosales	Wood	Munessa-Shashemen	Ethiopia	7.22	38.62	L1
[98]	Soil				Oregon	US	45.05	-123.95	L4
[99]	Soil				Åheden	Sweden	64.23	19.77	L5
[100]	3 species [m]	Poaceae<br>Fabaceae<br>Scrophulariaceae	Poales<br>Fabales<br>Scrophulariales	Grass<br>Forb	Zanjan	Iran	36	48	L3
[101]	*Veronica rechingeri*	Scrophulariaceae	Scrophulariales	Forb	Zanjan	Iran	36.67	47.33	L3

Table S1 Details of insufficiently identified ITS data source used in this study (continued from previous pages)

Source Reference	Biomes				
	Ecosystem	Climate	Biogeographical realm	Continent	Pangeae
[1]	Anthropogenic	Warm temperate	Palearctic	Europe	Laurasia
[2]	Anthropogenic	Snow	Nearctic	North America	Laurasia
[3]	Grassland	Warm temperate	Palearctic	Europe	Laurasia
[4]	Successional	Arid	Nearctic	North America	Laurasia
[5]	Successional	Warm temperate	Palearctic	Europe	Laurasia
[6]	Grassland	Warm temperate	Palearctic	Europe	Laurasia
[7]	Successional	Snow	Palearctic	Europe	Laurasia
[7]-1	Successional	Warm temperate	Palearctic	Europe	Laurasia
[8]	Grassland	Arid	Neotropic	South America	Gondwana
[9]	Grassland	Arid	Palearctic	Europe	Laurasia
[10]	Successional	Warm temperate	Palearctic	Europe	Laurasia
[11]	Grassland	Arid	Nearctic	North America	Laurasia
[12]	Anthropogenic	Warm temperate	Nearctic	North America	Laurasia
[13]	Forest	Arid	Australasia	Oceania	Gondwana
[14]	Forest	Arid	Australasia	Oceania	Gondwana
[15]	Successional	Arid	Nearctic	North America	Laurasia
[16]	Anthropogenic	Warm temperate	Palearctic	Europe	Laurasia
[17]	Forest	Arid	Australasia	Oceania	Gondwana
[18]	Shrubland	Arid	Afrotropic	Africa	Gondwana
[19]	Anthropogenic	Warm temperate	Palearctic	Europe	Laurasia

[20]	Forest	Arid	Australasia	Oceania	Gondwana
[21]	Successional	Snow	Nearctic	North America	Laurasia
[22]	Successional	Warm temperate	Palearctic	Europe	Laurasia
[23]	Anthropogenic	Warm temperate	Palearctic	Europe	Laurasia
[24]	Grassland	Warm temperate	Palearctic	Europe	Laurasia
[25]	Anthropogenic	Warm temperate	Palearctic	Europe	Laurasia
[26]	Forest	Warm temperate	Nearctic	North America	Laurasia
[27]	Forest	Snow	Nearctic	North America	Laurasia
[28]	Forest	Equatorial	Neotropic	South America	Gondwana
[29]	Forest	Warm temperate	Palearctic	Europe	Laurasia
[30]	Grassland	Warm temperate	Palearctic	Europe	Laurasia
[31]	Anthropogenic	Warm temperate	Palearctic	Europe	Laurasia
[31]-1	Anthropogenic	Warm temperate	Palearctic	Europe	Laurasia
[32]	Anthropogenic	Warm temperate	Palearctic	Europe	Laurasia
[33]	Grassland	Arid	Nearctic	North America	Laurasia
[34]	Grassland	Warm temperate	Palearctic	Europe	Laurasia
[35]	Forest	Warm temperate	Nearctic	North America	Laurasia
[36]	Forest	Warm temperate	Nearctic	North America	Laurasia
[37]	Grassland	Warm temperate	Palearctic	Europe	Laurasia
[38]	Grassland	Arid	Nearctic	North America	Laurasia
[39]	Grassland	Warm temperate	Palearctic	Europe	Laurasia
[40]	Forest	Snow	Palearctic	Europe	Laurasia
[41]	Forest	Warm temperate	Palearctic	Europe	Laurasia
[42]	Grassland	Warm temperate	Palearctic	Europe	Laurasia
[43]	Forest	Snow	Nearctic	North America	Laurasia
[44]	Successional	Warm temperate	Palearctic	Europe	Laurasia

[45]	Grassland	Warm temperate	Palearctic	Europe	Laurasia
[46]	Anthropogenic	Arid	Afrotropic	Africa	Gondwana
[47]	Unknown	Warm temperate	Nearctic	North America	Laurasia
[48]	Successional	Warm temperate	Palearctic	Asia	Laurasia
[49]	Successional	Warm temperate	Palearctic	Asia	Laurasia
[50]	Successional	Warm temperate	Palearctic	Europe	Laurasia
[51]	Successional	Warm temperate	Palearctic	Europe	Laurasia
[52]	Forest	Warm temperate	Nearctic	North America	Laurasia
[53]	Successional	Polar	Palearctic	Europe	Laurasia
[54]	Successional	Polar	Palearctic	Europe	Laurasia
[55]	Anthropogenic	Warm temperate	Palearctic	Europe	Laurasia
[56]	Grassland	Warm temperate	Palearctic	Europe	Laurasia
[57]	Grassland	Arid	Neotropic	South America	Gondwana
[58]	Forest	Warm temperate	Nearctic	North America	Laurasia
[59]	Forest	Arid	Nearctic	North America	Laurasia
[60]	Forest	Warm temperate	Palearctic	Europe	Laurasia
[61]	Anthropogenic	Warm temperate	Nearctic	North America	Laurasia
[62]	Anthropogenic	Warm temperate	Palearctic	Europe	Laurasia
[63]	Anthropogenic	Warm temperate	Nearctic	North America	Laurasia
[64]	Grassland	Warm temperate	Palearctic	Europe	Laurasia
[65]	Grassland	Warm temperate	Palearctic	Europe	Laurasia
[66]	Grassland	Warm temperate	Palearctic	Europe	Laurasia
[67]	Successional	Warm temperate	Australasia	Oceania	Gondwana
[68]	Anthropogenic	Warm temperate	Palearctic	Europe	Laurasia
[69]	Grassland	Warm temperature	Palearctic	Europe	Laurasia
[70]	Grassland	Warm temperate	Palearctic	Europe	Laurasia

[71]	Anthropogenic	Warm temperate	Palearctic	Europe	Laurasia
[72]	Anthropogenic	Equatorial	Neotropic	South America	Gondwana
[73]	Anthropogenic	Equatorial	Indo-Malay	Asia	Gondwana
[74]	Grassland	Equatorial	Neotropic	Nouth America	Laurasia
[75]	Grassland	Equatorial	Neotropic	Nouth America	Laurasia
[76]	Unknown	Snow	Nearctic	North America	Laurasia
[77]	Anthropogenic	Warm temperate	Palearctic	North America	Laurasia
[78]	Forest	Warm temperate	Palearctic	Europe	Laurasia
[79]	unknown	Warm temperate	Palearctic	Europe	Laurasia
[80]	Anthropogenic	Warm temperate	Nearctic	North America	Laurasia
[81]	Unknown	Arid	Nearctic	North America	Laurasia
[82]	Unknown	Warm temperate	Palearctic	Europe	Laurasia
[83]	Forest	Polar	Nearctic	North America	Laurasia
[84]	Forest	Polar	Nearctic	North America	Laurasia
[85]	Grassland	Equatorial	Afrotropic	Africa	Gondwana
[86]	Unknown	Warm temperate	Palearctic	Europe	Laurasia
[87]	Anthropogenic	Arid	Afrotropic	Africa	Gondwana
[88]	Anthropogenic	Arid	Afrotropic	Africa	Gondwana
[89]	Forest	Arid	Nearctic	North America	Laurasia
[90]	Forest	Arid	Nearctic	North America	Laurasia
[91]	Successional	Warm temperate	Palearctic	Europe	Laurasia
[91]-1	Successional	Warm temperate	Palearctic	Europe	Laurasia
[92]	Successional	Equatorial	Neotropic	South America	Gondwana
[93]	Grassland	Arid	Nearctic	North America	Laurasia
[94]	Successional	Warm temperate	Palearctic	Europe	Laurasia
[95]	Anthropogenic	Warm temperate	Nearctic	North America	Laurasia

[96]	Forest	Warm temperate	Palearctic	Europe	Laurasia
[97]	Forest	Arid	Afrotropic	Africa	Gondwana
[98]	Forest	Warm temperate	Nearctic	North America	Laurasia
[99]	Forest	Warm temperate	Palearctic	Europe	Laurasia
[100]	Anthropogenic	Arid	Palearctic	Asia	Laurasia
[101]	Anthropogenic	Arid	Palearctic	Asia	Laurasia

[a] *Agrostis stolonifera; Agrostis scabra; Dichanthelium lanuginosum*

[b] *Plantago major;Plantago lanceolata;Briza media;Arrhenatherum elatius;Taraxacum officinale;Dactylis glomerata;Potentilla erecta;Lychnis flos-cuculi;Trifolium repens;Trifolium pratense*

[c] *Zea mays ;Inula conyzae; Tragopogon pratensis;Vicia sativa;Torilis japonica; Vicia hirsuta; Galium aparine; Epilobium ciliatum; Festuca rupicola; Poa pratensis; Elymus repens; Arrhenatherum elatius;Arum maculatum; Artemisia vulgaris*

[d] *Puccinellia distans; Dactylis glomerata; Cardamine pratensis; Trifolium pretense; Ranunculus ficaria; Arum maculatum; Galium aparine; Vicia hirsute; Vicia sativa; Vicia tetrasperma; Epilobium ciliatum; Festuca pratensis; Artemisia vulgaris; Clematis vitalba*

[e] *Inga edulis; Calliandra thyrsifolia; Schizolobium sp.; Cedrela odorata; Inga paterno*

[f] *Calliandra thyrsifolia;Hyeronima oblonga;Tabebuia ochracea;Inga paterno*

[g] *Inula salicin; Bromus erectus; Origanum vulgare; Medicago sativa*

[h] *Gentiana acaulis; Gentiana verna; Crocus albiflorus;Thymus pulegioides; Leontodon hispidus; Trifolium sp.; Ranunculus montanus; Hieracium hoppeanum; Polygala vulgaris*

[i] *Stipagrostis ciliata; Stipagrostis hochstetteriana; Stipagrostis namaquensis; Acacia mellifera*

[j] *Aristida cf. Meridionalis; Eragrostis cf. Lehmanniana; Schmidtia cf. Pappophoroides; Chloris sp.; Stipagrostis cf. Ciliata; Hyperthelia dissoluta; Eragrostis cf. echinochloidea*

[k] *Aster tripolium; Puccinellia maritima; Salicornia europaea*

[l] *Lycopodium clavatum var. Clavatum; Lycopodium clavatum ssp. Contigum; Huperzia crassa ssp. Crassa; Huperzia tetragona; Huperzia affinis; Huperzia urbanii; Huperzia hypogaea*

[m] *Triticum aestivum; Medicago sativa; Veronica orientalis*

1. Alie I, Wiemken A, Redecker D: **Molecular identification of arbuscular mycorrhizal fungi in roots from natural and agricultural sites.** *unpublished*.
2. Amend AS, Seifert KA, Bruns TD: **Spiking pyrosequencing runs with known spore quantities reveals low error rates and reasonable semiquantitative results for fungal community analyses.** *unpublished*.
3. Anderson IC, Campbell CD, Prosser JI: **Potential bias of fungal 18S rDNA and internal transcribed spacer polymerase chain reaction primers for estimating fungal biodiversity in soil.** *Environmental microbiology* 2003, **5:**36-47.
4. Appoloni S. Symbiosis in extreme environments: Molecular community analysis of arbuscular mycorrhizal fungi in roots of geothermal soils in Yellowstone National Park (USA) [Basel: University of Basel, Switzerland.; 2006.
5. Artz RRE, Anderson IC, Chapman SJ, Hagn A, Schloter M, Potts JM, Campbell CD: **Changes in fungal community composition in response to vegetational succession during the natural regeneration of cutover peatlands.** *Microbial ecology* 2007, **54:**508-522.
6. Börstler B, Renker C, Kahmen A, Buscot F: **Species composition of arbuscular mycorrhizal fungi in two mountain meadows with differing management types and levels of plant biodiversity.** *Biology and Fertility of Soils* 2006, **42:**286-298.
7. Baar J, Paradi I, Lucassen EC, Hudson-Edwards KA, Redecker D, Roelofs JGM, Smolders AJP: **Molecular analysis of AMF diversity in aquatic macrophytes: A comparison of oligotrophic and utra-oligotrophic lakes.** *Aquatic Botany* 2010.
8. Bidartondo MI, Redecker D, Hijri I, Wiemken A, Bruns TD, Domínguez L, Sérsic A, Leake JR, Read DJ: **Epiparasitic plants specialized on arbuscular mycorrhizal fungi.** *Nature* 2002, **419:**389-392.
9. Błaszkowski J, Blanke V, Renker C, Buscot F: **Glomus aurantium and G. xanthium, new species in Glomeromycota.** *Mycotaxon* 2004, **90:**447-467.
10. Blaszkowski J, Ryszka P, Oehl F, Koegel S, Wiemken A, Kovacs G, Redecker D: **Glomus sp. FO128-131 and G. bistratum, two new species of arbuscular mycorrhizal fungi (Glomeromycota) found in maritime sand dunes.** *unpublished*.
11. Blosser GD: **Multiple techniques establish arbuscular mycorrhizae in northern California vernal pools: Islands of endemism in grasslands dominated by exotic plant species.** *unpublished*.
12. Bonito G, Isikhuemhen OS, Vilgalys R: **Identification of fungi associated with municipal compost using DNA-based techniques.** *Bioresource technology* 2010, **101:**1021-1027.
13. Bougoure DS, Cairney JWG: **Assemblages of ericoid mycorrhizal and other root - associated fungi from Epacris pulchella (Ericaceae) as determined by culturing and direct DNA extraction from roots.** *Environmental Microbiology* 2005, **7:**819-827.
14. Bougoure DS, Parkin PI, Cairney JWG, Alexander IANJ, Anderson IANC: **Diversity of fungi in hair roots of Ericaceae varies along a vegetation gradient.** *Molecular Ecology* 2007, **16:**4624-4636.

15. Bunn R, Lekberg Y, Zabinski C: **Arbuscular mycorrhizal fungi ameliorate temperature stress in thermophilic plants.** *Ecology* 2009, **90:**1378-1388.
16. Ceccarelli N, Curadi M, Martelloni L, Sbrana C, Picciarelli P, Giovannetti M: **Mycorrhizal colonization impacts on phenolic content and antioxidant properties of artichoke leaves and flower heads two years after field transplant.** *Plant and soil* 2010, **335:**311-323.
17. Chen DM, Cairney JWG: **Investigation of the influence of prescribed burning on ITS profiles of ectomycorrhizal and other soil fungi at three Australian sclerophyll forest sites.** *Mycological research* 2002, **106:**532-540.
18. Cloete K, Valentine A, Blomerus L, Botha A, Pèrez-Fernández M: **Nutritional effects of indigenous arbuscular mycorrhizal associations on the sclerophyllous species *Agathosma betulina*.** *Web Ecology* 2007, **77:**77-86.
19. Corradi N, Ruffner B, Croll D, Colard A, Horak A, Sanders IR: **High-level molecular diversity of copper-zinc superoxide dismutase genes among and within species of arbuscular mycorrhizal fungi.** *Applied and Environmental Microbiology* 2009, **75:**1970.
20. Curlevski NJA, Xu Z, Anderson I, Cairney JWG: **Diversity of soil and rhizosphere fungi under Araucaria bidwillii (Bunya pine) at an Australian tropical montane rainforest site.** *Fungal Diversity* 2010, **40:**12-22.
21. Deslippe JR, Simard SW, Mohn BW: **Long-term warming of arctic tundra selects for ectomycorrhizal fungi species with greater proteolytic capacity.** *unpublished*.
22. Fechner LC, Vincent-Hubert F: **Community fingerprinting of bacteria and eukaryotes from freshwater biofilms using automated ribosomal intergenic spacer analysis.** *unpublished*.
23. Galván GA, Parádi I, Burger K, Baar J, Kuyper TW, Scholten OE, Kik C: **Molecular diversity of arbuscular mycorrhizal fungi in onion roots from organic and conventional farming systems in the Netherlands.** *Mycorrhiza* 2009, **19:**317-328.
24. Gamper HA, Walker C, Schüßler A: **Diversispora celata sp. nov: molecular ecology and phylotaxonomy of an inconspicuous arbuscular mycorrhizal fungus.** *New Phytologist* 2009, **182:**495-506.
25. Gosling P, Proctor M, Jones J, Bending GD: **Arbuscular mycorrhizal fungi of the genus Paraglomus show sporadic distribution, and low diversity in agricultural soils.** *unpublished*.
26. Hanson CA, Allison SD, Bradford MA, Wallenstein MD, Treseder KK: **Fungal taxa target different carbon sources in forest soil.** *Ecosystems* 2008, **11:**1157-1167.
27. Hartmann M, Lee S, Chapman WK, Hallam SJ, Mohn WW: **Timber harvesting affects bacterial, archaeal and eukaryal communities throughout the soil depth profile.** *unpublished*.
28. Haug I, Lempe J, Homeier J, Weiß M, Setaro S, Oberwinkler F, Kottke I: **Graffenrieda emarginata (Melastomataceae) forms mycorrhizas with Glomeromycota**

and with a member of the Hymenoscyphus ericae aggregate in the organic soil of a neotropical mountain rain forest. *Canadian Journal of Botany* 2004, **82:**340-356.

29. Hedh J, Wallander H, Erland S: **Ectomycorrhizal mycelial species composition in apatite amended and non-amended mesh bags buried in a phosphorus-poor spruce forest.** *Mycological Research* 2008, **112:**681-688.
30. Hempel S, Renker C, Buscot F: **Differences in the species composition of arbuscular mycorrhizal fungi in spore, root and soil communities in a grassland ecosystem.** *Environmental microbiology* 2007, **9:**1930-1938.
31. Hijri I, Sýkorová Z, Oehl F, Ineichen K, Mäder P, Wiemken A, Redecker D: **Communities of arbuscular mycorrhizal fungi in arable soils are not necessarily low in diversity.** *Molecular Ecology* 2006, **15:**2277-2289.
32. Hijri I, Sykorova Z, Oehl F, Ineichen K, Maeder P, Wiemken A, Redecker D: **It's not the ploughing: communities of arbuscular mycorrhizal fungi in arable soils are not necessarily low in diversity.** *unpublished*.
33. Hollister EB, Boutton TW, Schadt CW, Palumbo AV: **Influence of plant functional types on soil microbial community composition in a temperate savanna.** *unpublished*.
34. Jean-Claude Abadie JCA, Püttsepp ÜPÜ, Gebauer GGG, Faccio AFA, Bonfante PBP, Marc-André Selosse MAS: **Cephalanthera longifolia (Neottieae, Orchidaceae) is mixotrophic: a comparative study between green and nonphotosynthetic individuals.** *Botany* 2006, **84:**1462-1477.
35. Jumpponen A, Jones K: **Massively parallel 454-sequencing indicates hyperdiverse fungal communities in temperate Quercus macrocarpa phyllosphere.** *New Phytologist* 2009, **184:**438-448.
36. Jumpponen A, JONES KL, David Mattox J, YAEGE C: **Massively parallel 454-sequencing of fungal communities in Quercus spp. ectomycorrhizas indicates seasonal dynamics in urban and rural sites.** *Molecular Ecology* 2010, **19:**41-53.
37. König S, Wubet T, Dormann CF, Hempel S, Renker C, Buscot F: **TaqMan real-time PCR assays to assess arbuscular mycorrhizal responses to field manipulation of grassland biodiversity: Effects of soil characteristics, plant species richness, and functional traits.** *Applied and environmental microbiology* 2010, **76:**3765-3775.
38. Khidir H, Eudy D, Porras-Alfaro A, Herrera J, Natvig D, Sinsabaugh R: **A general suite of fungal endophytes dominate the roots of two dominant grasses in a semiarid grassland.** *Journal of Arid Environments* 2010, **74:**35-42.
39. Koenig S, Wubet T, Renker C, Buscot F: **Real-time PCR as a tool for easy and fast identification of arbuscular mycorrhizal fungal diversity in soil: to sequence or not to sequence, that is the question.** *unpublished*.
40. Korkama T, Fritze H, Pakkanen A, Pennanen T: **Interactions between extraradical ectomycorrhizal mycelia, microbes associated with the mycelia and growth**

rate of Norway spruce (Picea abies) clones. *New Phytologist* 2007, **173:**798-807.

41. Kovács GM, Balázs T, Pénzes Z: **Molecular study of arbuscular mycorrhizal fungi colonizing the sporophyte of the eusporangiate rattlesnake fern (Botrychium virginianum, Ophioglossaceae).** *Mycorrhiza* 2007, **17:**597-605.
42. Krüger M, Stockinger H, Krüger C, Schüßler A: **DNA-based species level detection of Glomeromycota: one PCR primer set for all arbuscular mycorrhizal fungi.** *New Phytologist* 2009, **183:**212-223.
43. Lamarche J, Seguin A, Hamelin RC: **Changes in soil fungal communities in the presence of genetically modified white spruce transformed with *Trichoderma harzianum* endochitinase gene (ech42).** *unpublished*.
44. Landwehr M, Hildebrandt U, Wilde P, Nawrath K, Tóth T, Biró B, Bothe H: **The arbuscular mycorrhizal fungus Glomus geosporum in European saline, sodic and gypsum soils.** *Mycorrhiza* 2002, **12:**199-211.
45. Lang C, Weisse J, Polle A: **Characterization of arbuscular mycorrhiza on Fraxinus excelsior and Acer spp. in the National Park Hainich, Thuringa, Germany.** *unpublished*.
46. Lekberg Y, Koide RT, Rohr JR, ALDRICH ‐ WOLFE L, Morton JB: **Role of niche restrictions and dispersal in the composition of arbuscular mycorrhizal fungal communities.** *Journal of Ecology* 2007, **95:**95-105.
47. Mulbry W, Millner P, Reynolds S: **Design of species specific probes for Entrophospora contigua.** *unpublished*.
48. Nagano Y, Nagahama T, Hatada Y, Nunoura T, Takami H, Miyazaki J, Takai K, Horikoshi K: **Fungal diversity in deep-sea sediments.** *unpublished*.
49. Nagano Y, Nagahama T, Hatada Y, Nunoura T, Takami H, Miyazaki J, Takai K, Horikoshi K: **Fungal diversity in deep-sea sediments–the presence of novel fungal groups.** *Fungal Ecology* 2010, **3:**316-325.
50. Napoli C, Mello A, Borra A, Vizzini A, Sourzat P, Bonfante P: **Tuber melanosporum, when dominant, affects fungal dynamics in truffle grounds.** *New Phytologist* 2010, **185:**237-247.
51. Neubert K, Mendgen K, Brinkmann H, Wirsel SGR: **Only a few fungal species dominate highly diverse mycofloras associated with the common reed.** *Applied and Environmental Microbiology* 2006, **72:**1118.
52. O'Brien HE, Parrent JL, Jackson JA, Moncalvo JM, Vilgalys R: **Fungal community analysis by large-scale sequencing of environmental samples.** *Applied and environmental microbiology* 2005, **71:**5544.
53. Oberkofler I, Kuhnert R, Peintner U: **Soil fungal communities producing high quantities of biomass in snow covered primary successional soils: Who are they?** *unpublished*.
54. Oberkofler I, Peintner U: **Winter active fungi in snow-covered alpine soil.** *unpublished*.

55. Oehl F, Blaszkowski J, Sykorova Z, Boller T, Wiemken A, Redecker D: ***Acaulospora sieverdingii*, a new arbuscular mycorrhizal fungus from European agricultural systems.** *unpublished*.

56. Oehl F, Sýkorová Z, Redecker D, Wiemken A, Sieverding E: **Acaulospora alpina, a new arbuscular mycorrhizal fungal species characteristic for high mountainous and alpine regions of the Swiss Alps.** *Mycologia* 2006, **98:**286-294.

57. Pagano MC, Oliveira CA, Gomes EA, Cabello MN, Persiano AC, Scotti MR: **Morphological and molecular characterization of glomalean mycorrhizal fungi in a semi-arid region of Minas Gerais, Brazil.** *unpublished*.

58. Parrent JL, Vilgalys R: **Biomass and compositional responses of ectomycorrhizal fungal hyphae to elevated CO_2 and nitrogen fertilization.** *New Phytologist* 2007, **176:**164-174.

59. Porras-Alfaro A, Herrera J, Sinsabaugh RL, Odenbach KJ, Lowrey T, Natvig DO: **Novel root fungal consortium associated with a dominant desert grass.** *Applied and Environmental Microbiology* 2008, **74:**2805.

60. Porter TM, Schadt CW, Rizvi L, Martin AP, Schmidt SK, Scott-Denton L, Vilgalys R, Moncalvo JM: **Widespread occurrence and phylogenetic placement of a soil clone group adds a prominent new branch to the fungal tree of life.** *Molecular Phylogenetics and Evolution* 2008, **46:**635-644.

61. Pringle A, Moncalvo JM, Vilgalys R: **High levels of variation in ribosomal DNA sequences within and among spores of a natural population of the arbuscular mycorrhizal fungus Acaulospora colossica.** *Mycologia* 2000, 259-268.

62. Redecker D, Hijri I, Wiemken A: **Molecular identification of arbuscular mycorrhizal fungi in roots: perspectives and problems.** *Folia Geobotanica* 2003, **38:**113-124.

63. Redecker D, Hijri M, Dulieu H, Sanders IR: **Phylogenetic analysis of a dataset of fungal 5.8 S rDNA sequences shows that highly divergent copies of internal transcribed spacers reported from Scutellospora castanea are of ascomycete origin.** *Fungal Genetics and Biology* 1999, **28:**238-244.

64. Renker C, Blanke V, Buscot F: **Diversity of arbuscular mycorrhizal fungi in grassland spontaneously developed on area polluted by a fertilizer plant.** *Environmental Pollution* 2005, **135:**255-266.

65. Renker C, Heinrichs J, Kaldorf M, Buscot F: **Combining nested PCR and restriction digest of the internal transcribed spacer region to characterize arbuscular mycorrhizal fungi on roots from the field.** *Mycorrhiza* 2003, **13:**191-198.

66. Renker C, Weißhuhn K, Kellner H, Buscot F: **Rationalizing molecular analysis of field-collected roots for assessing diversity of arbuscular mycorrhizal fungi: to pool, or not to pool, that is the question.** *Mycorrhiza* 2006, **16:**525-531.

67. Russell J, Bulman S: **The liverwort Marchantia foliacea forms a specialized symbiosis with arbuscular mycorrhizal fungi in the genus Glomus.** *New Phytologist* 2005, **165:**567-579.

68. Ryszka P, Turnau K, Anielska T, Goralska K, Waligorski P, Bialonska D, Jurkiewicz A: **Influence of mycorrhizal fungi on plant vitality and level of active compounds in *Arnica montana* L.** *unpublished*.

69. Sýkorová Z, Ineichen K, Wiemken A, Redecker D: **The cultivation bias: different communities of arbuscular mycorrhizal fungi detected in roots from the field, from bait plants transplanted to the field, and from a greenhouse trap experiment.** *Mycorrhiza* 2007, **18:**1-14.

70. Sýkorová Z, Wiemken A, Redecker D: **Cooccurring Gentiana verna and Gentiana acaulis and their neighboring plants in two Swiss upper montane meadows harbor distinct arbuscular mycorrhizal fungal communities.** *Applied and Environmental Microbiology* 2007, **73:**5426-5434.

71. Salehi JG, Akbari VS, Khayam NM, Zarei M, Soheilivand S, Karimi E, JM S: **Diversity of arbuscular mycorrhiza in wheat and barley fields in arid and semi-arid regions of Iran.** *unpublished*.

72. Scotti MR, Gomes E, Souza F: **Direct Submission Submitted (05-JAN-2010) Dept of Botany, UFMG, Av Antonio Carlos 6627, Belo Horizonte, MG 31270901, Brazil.** *unpublished*.

73. Sharma AK, Raab PA, Wiemken A, Adholeya A, Redecker D: **Contrasting effects of two G. coronatum ecotypes on growth and development of wheat (Triticum aestivum) and greengram (Vigna radiata).** *unpublished*.

74. Shepherd M, Jones ME, Carpenter FL: **Diversity of arbuscular mycorrhizal fungi subgroups colonizing the roots of Costa Rican rainforest trees.** *unpublished*.

75. Shepherd M, Nguyen L, Jones ME, Nichols JD, Carpenter FL: **A method for assessing arbuscular mycorrhizal fungi group distribution in tree roots by intergenic transcribed sequence variation.** *Plant and soil* 2007, **290:**259-268.

76. Sokolski S, Dalpe Y, Seguin S, Piche Y: **Parallel between molecular and morphological species within Glomus genus (Glomeromycota, Fungi): the case of protein-encoding genes.** *unpublished*.

77. St. Laurent A, Merwin IA, Thies JE: **Planting position and in-row orchard groundcover management strategies affect soil microbial community and apple replant disease severity.** *unpublished*.

78. Stefani FOP, Tanguay P, Pelletier G, Piché Y, Hamelin RC: **Impact of endochitinase-transformed white spruce on soil fungal biomass and ectendomycorrhizal symbiosis.** *Applied and Environmental Microbiology* 2010, **76:**2607-2614.

79. Stockinger H, Krüger M, Schüßler A: **DNA barcoding of arbuscular mycorrhizal fungi.** *New Phytologist* 2010, **187:**461-474.

80. Stockinger H, Walker C, Schüßler A: **'Glomus intraradices DAOM197198', a model fungus in arbuscular mycorrhiza research, is not Glomus intraradices.** *New Phytologist* 2009, **183:**1176-1187.

81. Sudarshana P, Claassen VP, Tyler BM: **High level of genetic diversity of the ribosomal RNA internal transcribed spacers among and within isolates of the Glomus intraradices species complex.** *unpublished*.

82. Szego D: **Direct Submission Submitted (05-JAN-2005) Szego D., Dept. of Plant Physiology, ELTE University, Pazmany Peter setany 1/C, H-1117, HUNGARY.** *unpublished*.
83. Taylor D, Booth MG, McFarland JW, Herriott IC, Lennon NJ, Nusbaum C, Marr TG: **Increasing ecological inference from high throughput sequencing of fungi in the environment through a tagging approach.** *Molecular Ecology Resources* 2008, **8:**742-752.
84. Taylor DL, Herriott IC, Long J, O'Neill K: **TOPO TA is A-OK: a test of phylogenetic bias in fungal environmental clone library construction.** *Environmental Microbiology* 2007, **9:**1329-1334.
85. Tchabi A, Burger S, Coyne D, Hountondji F, Lawouin L, Wiemken A, Oehl F: **Promiscuous arbuscular mycorrhizal symbiosis of yam (Dioscorea spp.), a key staple crop in West Africa.** *Mycorrhiza* 2009, **19:**375-392.
86. Thiéry O, Börstler B, Ineichen K, Redecker D: **Evolutionary dynamics of introns and homing endonuclease ORFs in a region of the large subunit of the mitochondrial rRNA in *Glomus* species (arbuscular mycorrhizal fungi, Glomeromycota).** *Molecular Phylogenetics and Evolution* 2010, **55:**599-610.
87. Uhlmann E, Görke C, Petersen A, Oberwinkler F: **Arbuscular mycorrhizae from semiarid regions of Namibia.** *Canadian Journal of Botany* 2004, **82:**645-653.
88. Uhlmann E, Gorke C, Petersen A, Oberwinkler F: **Arbuscular mycorrhizae from arid parts of Namibia.** *Journal of Arid Environments* 2006, **64:**221-237.
89. Waldrop MP, Zak DR, Blackwood CB, Curtis CD, Tilman D: **Resource availability controls fungal diversity across a plant diversity gradient.** *Ecology Letters* 2006, **9:**1127-1135.
90. Warren JM, Brooks JR, Meinzer FC, Eberhart JL: **Hydraulic redistribution of water from Pinus ponderosa trees to seedlings: evidence for an ectomycorrhizal pathway.** *New Phytologist* 2008, **178:**382-394.
91. Wilde P, Manal A, Stodden M, Sieverding E, Hildebrandt U, Bothe H: **Biodiversity of arbuscular mycorrhizal fungi in roots and soils of two salt marshes.** *Environmental Microbiology* 2009, **11:**1548-1561.
92. Winther JL, Friedman WE: **Arbuscular mycorrhizal associations in Lycopodiaceae.** *New Phytologist* 2008, **177:**790-801.
93. Winther JL, Friedman WE: **Subterranean plants: molecular identification of fungal networks and carbon flow.** *unpublished*.
94. Wirsel SGR: **Homogenous stands of a wetland grass harbour diverse consortia of arbuscular mycorrhizal fungi.** *FEMS microbiology ecology* 2004, **48:**129-138.
95. Wu T, Chellemi DO, Martin KJ, Graham JH, Rosskopf EN: **Discriminating the effects of agricultural land management practices on soil fungal communities.** *Soil Biology and Biochemistry* 2007, **39:**1139-1155.
96. Wubet T, Weiß M, Kottke I, Oberwinkler F: **Morphology and molecular diversity of arbuscular mycorrhizal fungi in wild and cultivated yew (Taxus baccata).** *Canadian Journal of Botany* 2003, **81:**255-266.

97. Wubet T, Weiß M, Kottke I, Teketay D, Oberwinkler F: **Molecular diversity of arbuscular mycorrhizal fungi in Prunus africana, an endangered medicinal tree species in dry Afromontane forests of Ethiopia.** *New Phytologist* 2004, **161:**517-528.

98. Yarwood SA, Bottomley PJ, Myrold DD: **Soil microbial communities associated with Douglas-fir and red alder stands at high-and low-productivity forest sites in Oregon, USA.** *Microbial Ecology* 2010, **60:**606-617.

99. Yarwood SA, Myrold DD, Högberg MN: **Termination of belowground C allocation by trees alters soil fungal and bacterial communities in a boreal forest.** *FEMS Microbiology Ecology* 2009, **70:**151-162.

100. Zarei M, Hempel S, Wubet T, Schaffer T, Koenig S, Salehi, Jouzani G, Saleh-Rastin N, Buscot F: **Diversity of arbuscular mycorrhizae in relation to soil properties and plant diversity along a gradient of heavy metal contamination.** *unpublished*.

101. Zarei M, König S, Hempel S, Nekouei MK, Savaghebi G, Buscot F: **Community structure of arbuscular mycorrhizal fungi associated to Veronica rechingeri at the Anguran zinc and lead mining region.** *Environmental Pollution* 2008, **156:**1277-1283.
