# Supplementary material for: Selectivity by host plants affects the distribution of arbuscular mycorrhizal fungi: evidence from ITS rDNA sequence metadata
Source: BMC Evol Biol. 2012 Apr 12;12:50. doi: 10.1186/1471-2148-12-50 (PMC3395829; doi:10.1186/1471-2148-12-50)
Supplement: Additional file 5 — Table S2. Details of sufficiently identified ITS (FIS) data source used in this study (PDF 190kb). [file 1471-2148-12-50-S5.PDF]

Table S2 Details of sufficiently identified ITS data source used in this study

Source Reference	AMF species	Country	Continent	Climate	Biogeographical realm	Latitude	Longitude	Latitude class	Pangaea
[60]-Rf87	*Glomus aggregatum*	Namibia	Africa	Arid	Afrotropic	-24	18	L2	Gondwana
[53]	*Glomus aggregatum*	Tunisia	Africa	Arid	Afrotropic	28	8	L2	Gondwana
[7]-Rf9	*Glomus aurantium*	Poland	Europe	Warm Temperate	Palearctic	53.42	14.59	L4	Laurasia
[54]-Rf79	*Glomus aurantium*	Switzerland	Europe	Warm Temperate	Palearctic	47.44	7.75	L4	Laurasia
[38]	*Glomus aureum*	Switzerland	Europe	Warm Temperate	Palearctic	46	5	L4	Laurasia
[38]	*Glomus badium*	Switzerland	Europe	Warm Temperate	Palearctic	46	5	L4	Laurasia
[16]	*Glomus caledonium*	Switzerland	Europe	Warm Temperate	Palearctic	46	8	L4	Laurasia
[53]	*Glomus caledonium*	UK	Europe	Warm Temperate	Palearctic				Laurasia
[20]-Rf37	*Glomus caledonium*	Germany	Europe	Warm Temperate	Palearctic	50.95	11.61	L4	Laurasia
[54]-Rf79	*Glomus caledonium*	Ecuator	South America	Equatorial	Neotropic	-1	-78	L1	Gondwana
[16]	*Glomus caledonium*	Switzerland	Europe	Warm Temperate	Palearctic	46	8	L4	Laurasia
[53]	*Glomus caledonium*	UK	Europe	Warm Temperate	Palearctic				Laurasia
[20]	*Glomus caledonium*	Germany	Europe	Warm Temperate	Palearctic	50.95	11.61	L4	Laurasia
[54] -Rf79	*Glomus caledonium*	UK	Europe	Warm Temperate	Palearctic	51.99	-0.63	L4	Laurasia
[53]	*Glomus cerebriforme*	Canada	North America	Snow	Nearctic	55	-106	L4	Laurasia

[53]	*Glomus cerebriforme*	Canada	North America	Snow	Nearctic				Laurasia
[10]-Rf6	*Glomus claroideum*	Germany	Europe	Warm Temperate	Palearctic	50.95	11.61	L4	Laurasia
[47]-Rf64	*Glomus claroideum*	Germany	Europe	Warm Temperate	Palearctic	51	11.67	L4	Laurasia
[65]-Rf101	*Glomus claroideum*	Iran	Asia	Warm Temperate	Palearctic	32	53	L3	Laurasia
[8]	*Glomus claroideum*	Germany	Europe	Warm Temperate	Palearctic	51	11.67	L4	Laurasia
[42]	*Glomus claroideum*	Denmark	Europe	Warm Temperate	Palearctic	55.47	9.17	L4	Laurasia
[53]	*Glomus claroideum*	Finland	Europe	Polar	Palearctic	61	25	L5	Laurasia
[60]-Rf87	*Glomus clarum*	Namibia	Africa	Arid	Afrotropic	-24	18	L2	Gondwana
[16]	*Glomus clarum*	Switzerland	Europe	Warm Temperate	Palearctic	46	8	L4	Laurasia
[5]-Rf8	*Glomus clarum*	Columbia	South America	Arid	Neotropic	-41.12	-71.392	L3	Gondwana
[53]	*Glomus clarum*	Cuba	North America	Equatorial	Nearctic	21	-77	L2	Laurasia
[13]-Rf31	*Glomus constrictum*	Germany	Europe	Warm Temperate	Palearctic	50.63	10.78	L4	Laurasia
[60]-Rf87	*Glomus constrictum*	Namibia	Africa	Arid	Afrotropic	-24	18	L2	Gondwana
[59]	*Glomus coronatum*	Italy	Europe	Arid	Palearctic	41	12	L3	Laurasia
[4]	*Glomus coronatum*	Italy	Europe	Arid	Palearctic	40.5	11.8	L3	Laurasia
[26]	*Glomus coronatum*	Italy	Europe	Arid	Palearctic	41	12	L3	Laurasia
[21]-Rf42	*Glomus coronatum*	Ecuador	South America	Equatorial	Neotropic	-1	-78	L1	Gondwana
[57]-Rf86	*Glomus coronatum*	India	Asia	Equatorial	Indo-Malay	20	78	L2	Gondwana

[53]	*Glomus custos*	Spain	Europe	Warm Temperate	Palearctic	40	-3	L3	Laurasia
[8]	*Glomus diaphanum*	US	North America	Warm Temperate	Nearctic	37	-95	L3	Laurasia
[53]	*Glomus diaphanum*	New Zealand	Oceania	Warm Temperate	Australasia	-40	174	L3	Gondwana
[26]	*Glomus dimorphicum*	Canada	North America	Snow	Nearctic	55	-106	L4	Laurasia
[12]-Rf24	*Glomus eburneum*	US	North America	Warm Temperate	Nearctic	37	-95	L3	Laurasia
[8]	*Glomus drummondi*	Poland	Europe	Warm Temperate	Palearctic	51	19	L4	Laurasia
[40]	*Glomus etunicatum*	US	North America	Arid	Nearctic	37	-95	L3	Laurasia
[54] -Rf79	*Glomus etunicatum*	Ethiopia	Africa	Arid	Afrotropic	9	40	L1	Gondwana
[26]	*Glomus fasciculatum*	UK	Europe	Warm Temperate	Palearctic	55	-3	L4	Laurasia
[46]	*Glomus ulvum*	Martinique	South America	Equatorial	Neotropic	14	-61	L1	Gondwana
[6]	*Glomus geosporum*	Denmark	Europe	Warm Temperate	Palearctic	56	9	L4	Laurasia
[23]-Rf44	*Glomus geosporum*	Hungary	Europe	Warm Temperate	Palearctic	47	19	L4	Laurasia
[22]	*Glomus geosporum*	Switzerland	Europe	Warm Temperate	Palearctic	46	8	L4	Laurasia
[62]-Rf91	*Glomus geosporum*	Slovenia	Europe	Warm Temperate	Palearctic	46	14	L4	Laurasia
[44]	*Glomus geosporum*	UK	Europe	Warm Temperate	Palearctic	55	-3	L4	Laurasia
[15]	*Glomus geosporum*	Germany	Europe	Warm Temperate	Palearctic	51	10	L4	Laurasia
[36]	*Glomus geosporum*	US	North America	Warm Temperate	Palearctic	37	-95	L3	Laurasia
[60] -Rf87	*Glomus heterosporum*	Namibia	Africa	Arid	Afrotropic	-24	18	L2	Gondwana
[56]-Rf69	*Glomus hoi*	Finland	Europe	Snow	Palearctic	61	25	L5	Laurasia

[63]-Rf91	*Glomus hoi*	Germany	Europe	Warm Temperate	Palearctic	51	10	L4	Laurasia
[6]	*Glomus intraradices*	Germany	Europe	Warm Temperate	Palearctic	53	10	L4	Laurasia
[9]	*Glomus intraradices*	US	North America	Equatorial	Nearctic	37	-95	L3	Laurasia
[9]	*Glomus intraradices*	Canada	North America	Snow	Nearctic	55	-106	L4	Laurasia
[9]	*Glomus intraradices*	Netherlands	Europe	Warm Temperate	Palearctic	52	5	L4	Laurasia
[9]	*Glomus intraradices*	Namibia	Africa	Arid	Afrotropic	-22	18	L2	Gondwana
[9]	*Glomus intraradices*	Japan	Asia	Warm Temperate	Palearctic	36	138	L3	Laurasia
[9]	*Glomus intraradices*	Switzerland	Europe	Warm Temperate	Palearctic	46	8	L4	Laurasia
[9]	*Glomus intraradices*	Australia	Oceania	Arid	Australasia	-25	133	L2	Gondwana
[9]	*Glomus intraradices*	Costa Rica	South America	Equatorial	Neotropic	9	-83	L1	Gondwana
[9]	*Glomus intraradices*	Kenya	Africa	Arid	Afrotropic	-0.02	37	L1	Gondwana
[6]	*Glomus irregulare*	Denmark	Europe	Warm Temperate	Palearctic	56	9	L4	Laurasia
[16]	*Glomus luteum*	Switzerland	Europe	Warm Temperate	Palearctic	46	8	L4	Laurasia
[21]-Rf42	*Glomus luteum*	Canada	North America	Snow	Nearctic	55	-106	L4	Laurasia
[46]	*Glomus megalocarpum*	Guadeloupe	South America	Equatorial	Neotropic	16	-62	L2	Gondwana
[53]	*Glomus microaggregatum*	Canada	North America	Snow	Nearctic	55	-106	L4	Laurasia
[37]	*Glomus monosporum*	France	Europe	Warm Temperate	Palearctic	46	2	L4	Laurasia
[50] -Rf66	*Glomus mosseae*	Germany	Europe	Warm Temperate	Palearctic	51	10	L4	Laurasia
[4]	*Glomus mosseae*	UK	Europe	Warm Temperate	Palearctic	55	-3	L4	Laurasia
[57]-Rf86	*Glomus mosseae*	Switzerland	Europe	Warm Temperate	Palearctic	46	8	L4	Laurasia

[65]-Rf101	*Glomus mosseae*	Iran	Asia	Arid	Palearctic	36.61	48.4	L4	Laurasia
[26]	*Glomus mosseae*	Finland	Europe	Polar	Palearctic	60.71	25.73	L5	Laurasia
[23]-Rf44	*Glomus mosseae*	Hungary	Europe	Warm Temperate	Palearctic	48.33	19.5	L4	Laurasia
[18]	*Glomus mosseae*	India	Asia	Equatorial	Indo-Malay	20.59	78.65	L2	Laurasia
[26]	*Glomus mosseae*	Philippine	Asia	Equatorial	Indo-Malay	14.63	121.05	L1	Gondwana
[26]	*Glomus mosseae*	Indonesia	Asia	Equatorial	Indo-Malay	14.63	121.05	L1	Gondwana
[4]	*Glomus mosseae*	US	North America	Warm Temperate	Nearctic	37	-95	L3	Laurasia
[53]	*Glomus mosseae*	Canada	North America	Snow	Nearctic	51.25	-85.33	L4	Laurasia
[26]	*Glomus mosseae*	Venezuela	South America	Equatorial	Neotropic	6.41	66.59	L1	Gondwana
[64]-Rf97	*Glomus mosseae*	Ethiopia	Africa	Arid	Afrotropic	9	40.5	L1	Gondwana
[4]	*Glomus mosseae*	Namibia	Africa	Arid	Afrotropic	-22.95	18.66	L2	Gondwana
[2]	*Glomus mosseae*	Australia	Oceania	Arid	Australasia	-34.91	138.6	L3	Gondwana
[53]	*Glomus multiforum*	Poland	Europe	Warm Temperate	Palearctic	51	19	L4	Laurasia
[55]-Rf80	*Glomus proliferum*	Guadeloupe	South America	Equatorial	Neotropic	16	-62	L2	Gondwana
[46]	*Glomus pulvinatum*	Martinique	South America	Equatorial	Neotropic	14	-61	L2	Gondwana
[5]-Rf8	*Glomus sinuosum*	US	North America	Arid	Nearctic	37	-95	L3	Laurasia
[47]	*Glomus versiforme*	Germany	Europe	Warm Temperate	Palearctic	51	11.67	L4	Laurasia
[21]-Rf42	*Glomus versiforme*	US	North America	Warm Temperate	Nearctic	37	-95	L3	Laurasia
[8]	*Glomus walkeri*	Poland	Europe	Warm Temperate	Palearctic	51	19	L4	Laurasia

[8]	*Glomus xanthium*	Poland	Europe	Warm Temperate	Palearctic	51	19	L4	Laurasia
[39]	*Acaulospora alpina*	Switzerland	Europe	Warm Temperate	Palearctic	46	8	L4	Laurasia
[28]	*Acaulospora colossica*	Brazil	South America	Equatorial	Neotropic	-22	-47	L2	Gondwana
[41]	*Acaulospora colossica*	US	North America	Arid	Nearctic	36	-78	L3	Laurasia
[43]	*Acaulospora denticulata*	Colombia	South America	Equatorial	Neotropic	4	-74	L1	Gondwana
[39]	*Acaulospora lacunosa*	US	North America	Warm Temperate	Palearctic	42	-70	L4	Laurasia
[21]	*Acaulospora laevis*	Australia	Oceania	Arid	Australasia	-33	149	L3	Gondwana
[54]	*Acaulospora laevis*	New Zealand	Oceania	Arid	Australasia	-32	151	L3	Gondwana
[43]	*Acaulospora mellea*	Brazil	South America	Equatorial	Neotropic	-21	-48	L2	Gondwana
[3]	*Acaulospora morrowiae*	US	North America	Warm Temperate	Nearctic	44.42	-110.61	L3	Laurasia
[43]	*Acaulospora morrowiae*	Brazil	South America	Equatorial	Neotropic	-25	-50	L2	Gondwana
[39]	*Acaulospora paulinae*	France	Europe	Warm Temperate	Palearctic	40	-1	L3	Laurasia
[21]	*Acaulospora cavernata*	UK	Europe	Warm Temperate	Palearctic	5	-1	L4	Laurasia
[45]	*Archaeospora trappei*	Namibia	Africa	Arid	Afrotropic	-9	20	L1	Gondwana
[45]	*Archaeospora trappei*	Austria	Europe	Warm Temperate	Palearctic	47	15	L4	Laurasia
[45]	*Archaeospora leptoticha*	US	North America	Warm Temperate	Nearctic	38	-122	L3	Laurasia
[10]-Rf6	*Archaeospora leptoticha*	Germany	Europe	Warm Temperate	Palearctic	50.95	11.16	L4	Laurasia

[56]-Rf69	*Archaeospora gerdemannii*	France	Europe	Warm Temperate	Palearctic	49.5	7.5	L4	Laurasia
[54]	*Diversispora spurca*	Germany	Europe	Warm Temperate	Palearctic	51	10	L4	Laurasia
[43]	*Entrophospora colombiana*	Colombia	South America	Equatorial	Neotropic	4	-74	L1	Gondwana
[31]	*Entrophospora contigua*	US	North America	Warm Temperate	Nearctic	38	-80	L3	Laurasia
[30]	*Entrophospora infrequens*	US	North America	Warm Temperate	Nearctic	40	-73	L3	Laurasia
[21]	*Entrophospora kentinensis*	China	Asia	Equatorial	Palearctic	22	120	L2	Laurasia
[56]	*Entrophospora schenckii*	Colombia	South America	Equatorial	Neotropic	4	-74	L1	Gondwana
[43]	*Gigaspora albida*	Colombia	South America	Equatorial	Neotropic	6	-80	L1	Gondwana
[32]	*Gigaspora albida*	Brazil	South America	Equatorial	Neotropic	-25	-50	L2	Gondwana
[29]	*Gigaspora decipiens*	Brazil	South America	Equatorial	Neotropic	-14	-51	L2	Gondwana
[43]	*Gigaspora decipiens*	Australia	Oceania	Arid	Australasia	-25	133	L2	Gondwana
[24]	*Gigaspora gigantea*	US	North America	Warm Temperate	Nearctic	37	-95	L3	Laurasia
[33]	*Gigaspora gigantea*	Panama	North America	Equatorial	Nearctic	8.5	-80	L1	Laurasia
[49]-Rf65	*Gigaspora rosea*	US	North America	Warm Temperate	Nearctic	30	-82	L2	Laurasia
[29]	*Gigaspora margarita*	Brazil	South America	Equatorial	Neotropic	-14	-51	L2	Gondwana

[17]	*Gigaspora margarita*	Switzerland	Europe	Warm Temperate	Palearctic	46	8	L4	Laurasia
[25]	*Gigaspora margarita*	New Zealand	Oceania	Arid	Australasia	-40	174	L3	Gondwana
[1]	*Gigaspora margarita*	Australia	Oceania	Arid	Australasia	-25	133	L2	Gondwana
[51]	*Gigaspora margarita*	Japan	Asia	Warm Temperate	Palearctic	36	138	L3	Laurasia
[61]	*Gigaspora margarita*	West Indices	North Amercia	Warm Temperate	Nearctic	20	-70	L2	Laurasia
[27]	*Paraglomus brasilianum*	US	North America	Warm Temperate	Nearctic	38	-80	L3	Laurasia
[48]	*Paraglomus laccatum*	Poland	Europe	Warm Temperate	Palearctic	53	15	L4	Laurasia
[19]	*Paraglomus laccatum*	Germany	Europe	Warm Temperate	Palearctic	50.24	11.60	L4	Laurasia
[34]	*Paraglomus occultum*	US	North America	Warm Temperate	Nearctic	44	-110	L4	Laurasia
[14]	*Scutellospora castanea*	France	Europe	Warm Temperate	Palearctic	46	2	L4	Laurasia
[52]	*Scutellospora cerradensis*	Japan	Asia	Warm Temperate	Palearctic	8.43	2.4	L1	Laurasia
[35]	*Scutellospora coralloidea*	US	North America	Warm Temperate	Nearctic	36.95	-120.1	L3	Laurasia
[58]	*Scutellospora fulgida*	Italy	Europe	Arid	Palearctic	43.1	11	L3	Laurasia
[11]	*Scutellospora reticulata*	Brazil	South America	Equatorial	Neotropic	-22.5	-43.8	L2	Gondwana

Note: Rf-xx represents the reference source here is the same with the reference in Table S1.

1. Antoniolli ZI, Schachtman D, Ophel-Keller K, Smith S: **Variation in rDNA ITS sequences in< i> Glomus mosseae</i> and< i> Gigaspora margarita</i> spores from a permanent pasture.** *Mycol Res* 2000, **104:**708-715.
2. Antoniolli ZI, Schachtman DP, Ophel-Keller K, Smith SE: **Variation in rDNA ITS sequences in Glomus mosseae and Gigaspora margarita spores from a permanent pasture.** *Mycol Res* 2000, **104:**708-715.
3. Appoloni S, Lekberg Y, Tercek MT, Zabinski CA, Redecker D: **Molecular community analysis of arbuscular mycorrhizal fungi in roots of geothermal soils in Yellowstone National Park (USA).** *Microbial ecology* 2008, **56:**649-659.
4. Avio L, Cristani C, Strani P, Giovannetti M: **Genetic and phenotypic diversity of geographically different isolates of Glomus mosseae.** *Can J Microbiol* 2009, **55:**242-253.
5. Bidartondo MI, Redecker D, Hijri I, Wiemken A, Bruns TD, Dominguez L, Sersic A, Leake JR, Read DJ: **Epiparasitic plants specialized on arbuscular mycorrhizal fungi.** *Nature* 2002, **419:**389-392.
6. Blaskowski J, Czerniawska B, Wubet T, Schafer T, Buscot F, Renker C: **Glomus irregulare, a new arbuscular mycorrhizal fungus in the Glomeromycota.** *Mycotaxon* 2008, **106:**247-267.
7. Błaszkowski J, Blanke V, Renker C, Buscot F: **Glomus aurantium and G. xanthium, new species in Glomeromycota.** *Mycotaxon* 2004, **90:**447-467.
8. Blaszkowski J, Renker C, Buscot F: **Glomus drummondii and G-walkeri, two new species of arbuscular mycorrhizal fungi (Glomeromycota).** *Mycol Res* 2006, **110:**555-566.
9. Borstler B, Raab PA, Thiery O, Morton JB, Redecker D: **Genetic diversity of the arbuscular mycorrhizal fungus Glomus intraradices as determined by mitochondrial large subunit rRNA gene sequences is considerably higher than previously expected.** *New Phytol* 2008, **180:**452-465.
10. Borstler B, Renker C, Kahmen A, Buscot F: **Species composition of arbuscular mycorrhizal fungi in two mountain meadows with differing management types and levels of plant biodiversity.** *Biol Fert Soils* 2006, **42:**286-298.
11. De Souza FA, Declerck S, Smit E, Kowalchuk GA: **Morphological, ontogenetic and molecular characterization of< i> Scutellospora reticulata (Glomeromycota)</i>.** *Mycological research* 2005, **109:**697-706.
12. Gamper HA, Walker C, Schussler A: **Diversispora celata sp nov: molecular ecology and phylotaxonomy of an inconspicuous arbuscular mycorrhizal fungus.** *New Phytol* 2009, **182:**495-506.
13. Hijri I, Sýkorová Z, Oehl F, Ineichen K, Mäder P, Wiemken A, Redecker D: **Communities of arbuscular mycorrhizal fungi in arable soils are not necessarily low in diversity.** *Molecular Ecology* 2006, **15:**2277-2289.
14. Hijri M, Kuhn G, Sanders IR: **Evidence for the evolution of multiple genomes in arbuscular mycorrhizal fungi.** *unpublished*.

15. Hildebrandt U, Janetta K, Bothe H: **Cloning of the ITS region from the AM fungus Glomus geosporum.** *unpublished*.
16. Jansa J, Mozafar A, Anken T, Ruh R, Sanders IR, Frossard E: **Diversity and structure of AMF communities as affected by tillage in a temperate soil.** *Mycorrhiza* 2002, **12:**225-234.
17. Jansa J, Mozafar A, Anken T, Ruh R, Sanders IR, Frossard E: **Isolation and identification of arbuscular mycorrhizal (AM) fungi from Swiss agricultural soils subjected to different tillage.** *unpublished*.
18. Jones NP, Krishnaraj PU, Pranav C, Kulkarni JH, Alagawadi AR: **Modified technique for PCR amplification and identification using single mycorrhizal spore.** *unpublished*.
19. König S, Wubet T, Dormann CF, Hempel S, Renker C, Buscot F: **TaqMan real-time PCR assays to assess arbuscular mycorrhizal responses to field manipulation of grassland biodiversity: Effects of soil characteristics, plant species richness, and functional traits.** *Applied and environmental microbiology* 2010, **76:**3765-3775.
20. Konig S, Wubet T, Dormann CF, Hempel S, Renker C, Buscot F: **TaqMan Real-Time PCR Assays To Assess Arbuscular Mycorrhizal Responses to Field Manipulation of Grassland Biodiversity: Effects of Soil Characteristics, Plant Species Richness, and Functional Traits.** *Appl Environ Microb* 2010, **76:**3765-3775.
21. Kruger M, Stockinger H, Kruger C, Schussler A: **DNA-based species level detection of Glomeromycota: one PCR primer set for all arbuscular mycorrhizal fungi.** *New Phytol* 2009, **183:**212-223.
22. Kuhn G, Hijri M, Sanders IR: **Evidence for the evolution of multiple genomes in arbuscular mycorrhizal fungi.** *unpublished*.
23. Landwehr M, Hildebrandt U, Wilde P, Nawrath K, Toth T, Biro B, Bothe H: **The arbuscular mycorrhizal fungus *Glomus geosporum* in European saline, sodic and gypsum soils.** *Mycorrhiza* 2002, **12:**199-211.
24. Lanfranco L, Bianciotto V, Lumini E, Souza M, Morton JB, Bonfante P: **A combined morphological and molecular approach to characterize isolates of arbuscular mycorrhizal fungi in Gigaspora (Glomales).** *unpublished*.
25. Lanfranco L, Delpero M, Bonfante P: **Intrasporal variability of ribosomal sequences in the endomycorrhizal fungus Gigaspora margarita.** *Molecular Ecology* 1999, **8:**37-45.
26. LloydMacgilp SA, Chambers SM, Dodd JC, Fitter AH, Walker C, Young JPW: **Diversity of the ribosomal internal transcribed spacers within and among isolates of Glomus mosseae and related mycorrhizal fungi.** *New Phytol* 1996, **133:**103-111.
27. Millner P, Mulbry W, Reynolds S: **Taxon - specific oligonucleotide primers for detection of two ancient endomycorrhizal fungi, Glomus occultum and Glomus brasilianum.** *FEMS microbiology letters* 2001, **196:**165-170.

28. Moreira-Souza M, Franco MC, Cardoso EJBN, Tsai SM, Moon DH: **Biodiversity of the mycorrhizal fungus Acaulospora collosica in tropical soils.** *unpublished*.
29. Moreira-Souza M, Gomes JE, Cardoso EJBN, Tsai SM: **Biodiversity of the arbuscular mycorrhizal fungi in tropical ecosystems.** *unpublished*.
30. Mulbry W, Millner P, Reynolds S: **Design of Species Specific DNA Probes for Entrophospora Species.** *unpublished*.
31. Mulbry W, Millner P, Reynolds S: **Design of species specific probes for Entrophospora contigua.** *unpublished*.
32. Mulbry W, Millner P, Reynolds S: **Design of species specific probes for Gigaspora albida.** *unpublished*.
33. Mulbry W, Millner P, Reynolds S: **Design of species specific probes for Gigaspora gigantea.** *unpublished*.
34. Mulbry W, Millner P, Reynolds S: **Design of species specific probes for Glomus occultum.** *unpublished*.
35. Mulbry W, Millner P, Reynolds S: **Design of species specific probes for Scutellospora coralloidea.** *unpublished*.
36. Mulbry W, Reynolds S, Millner P: **Design of species specific probes for *Glomus geosporum*.** *unpublished*.
37. Mulbry W, Reynolds S, Millner P: **Design of species specific probes for Glomus monosporum.** *unpublished*.
38. Oehl F, Redecker D, Sieverding E: **Glomus badium, a new sporocarpic mycorrhizal fungal species from European grasslands with higher soil pH.** *J Appl Bot & Food Qual* 2005, **79:**38-43.
39. Oehl F, Sýkorová Z, Redecker D, Wiemken A, Sieverding E: **Acaulospora alpina, a new arbuscular mycorrhizal fungal species characteristic for high mountainous and alpine regions of the Swiss Alps.** *Mycologia* 2006, **98:**286-294.
40. Pawlowska TE, Taylor JW: **Organization of genetic variation in individuals of arbuscular mycorrhizal fungi.** *Nature* 2004, **427:**733-737.
41. Pringle A, Moncalvo JM, Vilgalys R: **High levels of variation in ribosomal DNA sequences within and among spores of a natural population of the arbuscular mycorrhizal fungus Acaulospora colossica.** *Mycologia* 2000, 259-268.
42. Redecker D, Bruns TD: **Specific PCR primers to identify arbuscular mycorrhizal fungi (Glomales) within roots.** *unpublished*.
43. Redecker D, Hijri M, Dulieu H, Sanders IR: **Phylogenetic analysis of a dataset of fungal 5.8 S rDNA sequences shows that highly divergent copies of internal transcribed spacers reported from Scutellospora castanea are of ascomycete origin.** *Fungal Genet Biol* 1999, **28:**238-244.
44. Redecker D, Hijri M, Dulieu H, Sanders IR: **Phylogenetic analysis of a dataset of fungal 5.8S rDNA sequences shows that highly divergent copies of internal transcribed spacers reported from Scutellospora castanea are of ascomycete origin.** *Fungal Genet Biol* 1999, **28:**238-244.
45. Redecker D, Morton JB, Bruns TD: **Ancestral lineages of arbuscular mycorrhizal fungi (Glomales).** *Mol Phylogenet Evol* 2000, **14:**276-284.
46. Redecker D, Raab P, Oehl F, Camacho FJ, Courtecuisse R: **A novel clade of sporocarp-forming species of glomeromycotan fungi in the Diversisporales lineage.** *Mycol Prog* 2007, **6:**35-44.
47. Renker C, Blanke V, Buscot F: **Diversity of arbuscular mycorrhizal fungi in grassland spontaneously developed on area polluted by a fertilizer plant.** *Environ*

Pollut 2005, **135:**255-266.

48. Renker C, Blaszkowski J, Buscot F: **Paraglomus laccatum comb. nov.-a new member of Paraglomeraceae (Glomeromycota).** *Nova Hedwigia, 84* 2007, **3:**395-407.
49. Renker C, Heinrichs J, Kaldorf M, Buscot F: **Combining nested PCR and restriction digest of the internal transcribed spacer region to characterize arbuscular mycorrhizal fungi on roots from the field.** *Mycorrhiza* 2003, **13:**191-198.
50. Renker C, Weißhuhn K, Kellner H, Buscot F: **Rationalizing molecular analysis of field-collected roots for assessing diversity of arbuscular mycorrhizal fungi: to pool, or not to pool, that is the question.** *Mycorrhiza* 2006, **16:**525-531.
51. Sawaki H, Shinozaki N, Oyaizu H, Saito M: **PCR primer for detection of a new taxonomic group of glomalean fungi.** *unpublished*.
52. Sawaki H, Shinozaki N, Oyaizu H, Saito M: **PCR primer for detection of a new taxonomic group of glomalean fungi.** *unpublished*.
53. Sokolski S, Dalpe Y, Seguin S, Khasa D, Levesque CA, Piche Y: **Conspecificity of DAOM 197198, the model arbuscular mycorrhizal fungus, with Glomus irregulare: molecular evidence with three protein-encoding genes.** *Botany* 2010, **88:**829-838.
54. Stockinger H, Kruger M, Schussler A: **DNA barcoding of arbuscular mycorrhizal fungi.** *New Phytol* 2010, **187:**461-474.
55. Stockinger H, Walker C, Schüßler A: **'Glomus intraradices DAOM197198', a model fungus in arbuscular mycorrhiza research, is not Glomus intraradices.** *New Phytol* 2009, **183:**1176-1187.
56. Sykorova Z, Ineichen K, Wiemken A, Redecker D: **The cultivation bias: different communities of arbuscular mycorrhizal fungi detected in roots from the field, from bait plants transplanted to the field, and from a greenhouse trap experiment.** *Mycorrhiza* 2007, **18:**1-14.
57. Thiery O, Borstler B, Ineichen K, Redecker D: **Evolutionary dynamics of introns and homing endonuclease ORFs in a region of the large subunit of the mitochondrial rRNA in Glomus species (arbuscular mycorrhizal fungi, Glomeromycota).** *Mol Phylogenet Evol* 2010, **55:**599-610.
58. Turrini A, Avio L, Bedini S, Giovannetti M: **In situ collection of endangered arbuscular mychorrhizal fungi in a Mediterranean UNESCO Biosphere Reserve.** *Biodiversity and Conservation* 2008, **17:**643-657.
59. Turrini A, Sbrana C, Strani P, Pezzarossa B, Risaliti R, Giovannetti M: **A natural repository of arbuscular mycorrhizal fungi in Tuscan islands UNESCO biosphere reserve.** *unpublished*.
60. Uhlmann E, Görke C, Petersen A, Oberwinkler F: **Arbuscular mycorrhizae from semiarid regions of Namibia.** *Canadian Journal of Botany* 2004, **82:**645-653.
61. Waterman LD: **Sequence analysis of ribosomal DNA amplification products and investigations on protoplast isolation from vesicular-arbuscular mycorrhizal fungi of the genus Gigaspora.** *PhD Thesis* 1994.
62. Wilde P, Hildebrandt U, Backhausen S, Bothe H: ***Glomus geosporum* in a Slovenian salt marsh.** *unpublished*.

63. Wilde P, Manal A, Stodden M, Sieverding E, Hildebrandt U, Bothe H: **Biodiversity of arbuscular mycorrhizal fungi in roots and soils of two salt marshes.** *Environmental Microbiology* 2009, **11:**1548-1561.

64. Wubet T, Weiß M, Kottke I, Teketay D, Oberwinkler F: **Molecular diversity of arbuscular mycorrhizal fungi in Prunus africana, an endangered medicinal tree species in dry Afromontane forests of Ethiopia.** *New Phytol* 2004, **161:**517-528.

65. Zarei M, Konig S, Hempel S, Nekouei MK, Savaghebi G, Buscot F: **Community structure of arbuscular mycorrhizal fungi associated to Veronica rechingeri at the Anguran zinc and lead mining region.** *Environ Pollut* 2008, **156:**1277-1283.
